# iVS analysis to evaluate the impact of scaffold diversity in the binding to cellular targets relevant in cancer

**DOI:** 10.1080/14756366.2018.1518960

**Published:** 2018-10-26

**Authors:** Agostino Cilibrizzi, Giuseppe Floresta, Vincenzo Abbate, Maria Paola Giovannoni

**Affiliations:** a Institute of Pharmaceutical Science, King’s College London, London, UK;; b King’s Forensics, School of Population Health & Environmental Sciences, King’s College London, London, UK;; c Department of Drug Sciences, University of Catania, Catania, Italy;; d NEUROFARBA, Sezione di Farmaceutica e Nutraceutica, Università degli Studi di Firenze, Sesto Fiorentino, Italy

**Keywords:** Inverse virtual screening, heterocycles, small-molecules, scaffold diversity, biological targets

## Abstract

This study reports the application of inverse virtual screening (iVS) methodologies to identify cellular proteins as suitable targets for a library of heterocyclic small-molecules, with potential pharmacological implications. Standard synthetic procedures allow facile generation of these ligands showing a high degree of *core* scaffold diversity. Specifically, we have computationally investigated the binding efficacy of the new series for target proteins which are involved in cancer pathogenesis. As a result, nine macromolecules demonstrated efficient binding interactions for the molecular dataset, in comparison to the co-crystallised ligand for each target. Moreover, the iVS analysis led us to confirm that 27 analogues have high affinity for one or more examined cellular proteins. The additional evaluation of ADME and drug score for selected hits also highlights their capability as drug candidates, demonstrating valuable leads for further structure optimisation and biological studies.

## Graphical Abstract

**Figure:**
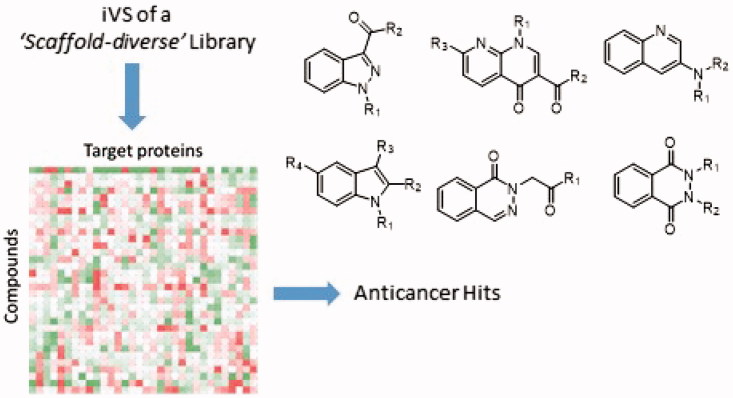

## 1. Introduction

Heterocyclic chemistry is one of the most valuable sources of novel molecules with a wide range of biological activities, mainly due to the unique ability of the resulting compounds to mimic the structure of endogenous ligands and reversibly bind to various targets of interest^1–3^. In medicinal chemistry, the main advantage of heterocyclic structures possibly rely on the capability of synthesising one such a library based on a specific *core*, allowing screening protocols against a variety of different targets^4^. Fused heterocycles can also be designed with almost unlimited combinations, resulting in novel bi- or polycyclic scaffolds with diverse physical, chemical, and biological properties. Overall, the fusion of rings leads to sterically well-defined and rigid structures, holding the promise for high functional specialisation which results from the ability to orient substituents in three-dimensional space as required by the biological targets^5^. From cancer therapy to the treatment of infectious, parasitic, and metabolic diseases, the drugs employed are often based on biologically active heterocyclic templates that interfere with the functioning of enzymes, the transmission of nerve impulses or the action of hormones on receptors, to name a few actions.

Scaffold diversity (i.e. variation of the nature of core scaffolds), appendage diversity (or building-block diversity, i.e. variation in structural moieties around a common scaffold), functional group diversity (i.e. variation in the functional groups present in the molecules) and stereochemical diversity (i.e. variation in the three-dimensional orientation of macromolecule-interacting residues) are the main determinants for late stage chemical diversification^6–8^. Indeed, it is generally recognised that the more structurally diverse a molecule is, the more likely it can interact with a particular biological macromolecule in a selective and specific manner^6^ ^,^ ^7^ ^,^ ^9–12^. In this context, the exploitation of scaffold diversity has a strategic role to reach amplified structural variation and explore new areas of the chemical space in biological investigations^9^ ^,^ ^13–15^. Moreover, it has been reported that the overall shape diversity of small-molecules is primarily dependent on the nature of the specific molecular scaffold (being the latter also intrinsically linked to functional diversity)^16^, with the peripheral substituents having a lower impact^17^ ^,^ ^18^. Therefore, there is a widespread consensus that the increase of scaffold diversity in a small-molecule library is one of the most effective means to implement its whole structural diversity^6^ ^,^ ^7^ ^,^ ^9^ ^,^ ^13–15^.

Computer-aided molecular screening has become a crucial tool in drug design and discovery and computational techniques represent a valid resource for the rapid evaluation of new compounds with potential biological activity. Currently, approaches such as structure-based, ligand-based and virtual screening are widely used in various drug discovery contexts, spanning from hit identification to lead optimisation stages^19–22^. The recognition of biological targets by synthetic molecules is of primary importance, as well as the possibility to analyse a big database of compounds by evaluating their binding mode with targets of pharmacological interest (i.e. virtual screening)^23^ ^,^ ^24^. In contrast, the inverse virtual screening (iVS) is a computational approach that focuses on the *in silico* evaluation of a panel of biological targets typically involved in diseases^25–28^. Specifically, multiple cellular proteins (from appropriately built databases) are screened by iVS in order to identify potential targets for suitable ligands of interest. This methodology allows the rapid analysis of crucial features in the process of hit identification, including target validation, drug repurposing and side effects/toxicity prediction. Moreover, iVS demonstrates a valuable tool to preliminary explore possible biological activities towards a selection of protein targets having pharmacological interest.

Herein we report the *in silico* investigation of 32 new heterocyclic small-molecules through iVS, in order to validate a scaffold-guided structural diversity approach for future biological tests. This compound dataset shows high variation in the nature of the *core* molecular scaffolds (i.e. indole, indazole, quinoline, naphtyridone, phthalazinone and phthalhydrazide). iVS analysis has been conducted through a panel of 32 selected proteins implicated in cancer progression and cancer cell survival^18^ ^,^ ^29^ ^,^ ^30^. The study highlights that the majority of compounds have potential to interact with the examined targets, representing an outstanding starting point to drive biological evaluation in a rapid and cost-effective fashion.

## 2. Results and discussion

### 2.1. Heterocyclic small-molecule dataset

The dataset of compounds is composed by 32 terms (Table 1) which have been easily obtained through standard synthetic methodologies (see Section 1, Supporting Information), in order to introduce (alkoxy)phenyl- and (halo)phenyl-based residues (typically recurrent in bioactive agents)^31–34^ in six heterocyclic scaffolds (i.e. indazole for **1a**–**f**, indole for **2a**–**h**, quinoline for **3a**–**d**, naphtyridone for **4a**–**j**, phthalazinone for **5** and phthalhydrazide **6a**–**d**; Table 1). The experimental procedures and characterisation data of all new intermediates and final compounds are reported in Supporting Information (Section 2).

**Table 1.** Structures of the heterocyclic small-molecules analysed by iVS screening.

### 2.2. Molecular modelling

The compound library was screened in iVS modality against a panel of 32 cellular targets (Table 1S, Supporting Information), which have been selected for their association to cancer progression and survival. This *in silico* approach allows the prediction of activity and selectivity through the evaluation of binding energies. Therefore, a large dataset of compounds can be narrowed to a defined group of promising candidates for following biological evaluation. For our purpose, calculations were performed with Autodock Vina, a validated software for iVS applications^29^ ^,^ ^30^. Docking analysis of crystallised ligands, with an established binding mode, were carried out in order to obtain a minimum energy level which has been used as the cut-off for the assessment of binding energies of the new ligands. In particular, the binding efficiency was evaluated through the ratio between the binding energies of analysed ligands and reference ligands co-crystallised in the protein, by applying Equation (1):

(1) $$\delta =\frac{\Delta \text{Gcompound}}{\Delta \text{Greference}\;\text{ligand}}$$

The values of binding energies have been organised in a matrix of 32 structures versus 32 selected cellular targets (as shown in Table 2S, Supporting Information). Each significant result was manually checked, to avoid odd or impossible interactions. From the library, compounds showing a δ ≥ 1 in a particular protein were selected and further analysed. A mathematical filter was also applied to the resulting energies as suggested by Bifulco et al.^29^ ^,^ ^30^, in order to overcome the lack of selectivity and occurrence of false positives, as well as to avoid systematic errors associated with the interaction of ligands and biological targets. Equation (2) was used to normalise the binding energy values in the matrix:

(2) $$V=V_{0}/\left[\left(M_{L}+M_{R}\right)/2\right]$$

**Table 2.** Results of calculated V values for the analysed biological targets in the study.

	Protein (PDB code)								
	3l3l	3oyw	4qmz	2fb8	3lbz	4ks8	4u5j	4ual	5h2u
Ligand^a^	0.818	0.569	0.930	1.040	0.739	0.828	0.803	1.046	1.077
**1a**	1.024	0.919	0.950	1.057	0.880	0.979	0.995	0.986	1.043
**1b**	0.963	0.939	0.956	1.040	0.861	1.020	0.989	0.969	1.037
**1c**	0.986	0.914	0.922	1.007	0.863	0.951	0.978	0.959	1.058
**1d**	0.987	0.755	0.922	1.053	0.900	1.058	1.014	1.016	0.996
**1e**	1.013	0.933	0.962	1.013	0.894	0.956	0.995	0.986	1.042
**1f**	0.983	0.782	1.047	1.058	0.910	1.063	1.054	1.076	1.086
**2a**	1.013	0.945	0.962	1.057	0.930	1.014	1.006	0.997	1.085
**2b**	0.955	0.955	0.983	1.044	0.914	1.048	1.015	0.994	1.073
**2c**	0.988	0.797	0.946	1.031	0.876	1.058	1.072	1.060	1.050
**2d**	0.949	0.909	0.964	1.036	0.858	0.946	0.951	0.999	1.065
**2e**	0.978	0.918	0.949	1.042	0.880	0.942	1.004	0.973	1.060
**2f**	1.017	0.928	0.991	1.080	0.890	0.996	0.989	1.034	1.077
**2g**	1.104	0.824	0.962	1.104	0.981	0.989	1.004	1.133	1.110
**2h**	1.109	0.909	0.940	1.055	1.007	1.037	1.040	1.008	1.083
**3a**	1.147	0.963	0.954	0.994	0.897	0.983	1.033	1.067	1.014
**3b**	1.151	0.953	0.993	0.964	0.926	0.951	0.967	1.059	1.006
**3c**	1.199	0.927	1.002	1.049	1.000	0.927	1.089	1.067	1.004
**3d**	1.169	0.874	1.002	1.060	0.912	0.973	0.999	1.090	1.036
**4a**	1.093	0.807	1.001	1.082	0.872	1.018	1.010	0.978	1.121
**4b**	1.078	0.884	1.010	1.067	0.884	1.027	1.030	1.053	1.148
**4c**	1.028	0.792	0.966	1.117	0.910	0.983	1.011	1.103	1.155
**4d**	1.053	0.784	1.041	1.141	0.889	0.987	1.095	1.016	1.146
**4e**	1.089	0.884	0.964	1.100	0.859	0.992	1.007	1.053	1.169
**4f**	1.109	0.866	1.030	1.043	0.929	0.977	1.015	1.006	1.136
**4g**	1.112	0.965	1.002	1.122	0.853	1.007	1.043	1.055	1.190
**4h**	1.023	0.863	0.984	0.944	0.967	0.965	1.028	0.961	1.074
**4j**	0.933	0.776	0.972	1.036	0.766	0.904	0.958	0.962	1.090
**5**	1.047	0.894	0.951	0.904	0.932	0.921	0.949	1.065	0.958
**6a**	1.104	0.820	0.954	1.093	0.923	1.006	0.987	1.067	1.057
**6b**	1.137	0.830	0.941	1.004	0.934	0.970	0.963	1.056	0.862
**6c**	1.137	0.878	1.005	1.051	0.891	0.964	1.024	1.113	1.132
**6d**	1.130	0.956	1.042	1.141	1.030	1.000	0.970	1.072	1.114

a co-crystallised ligand in the binding pocket of each protein.

**Figure 1.**
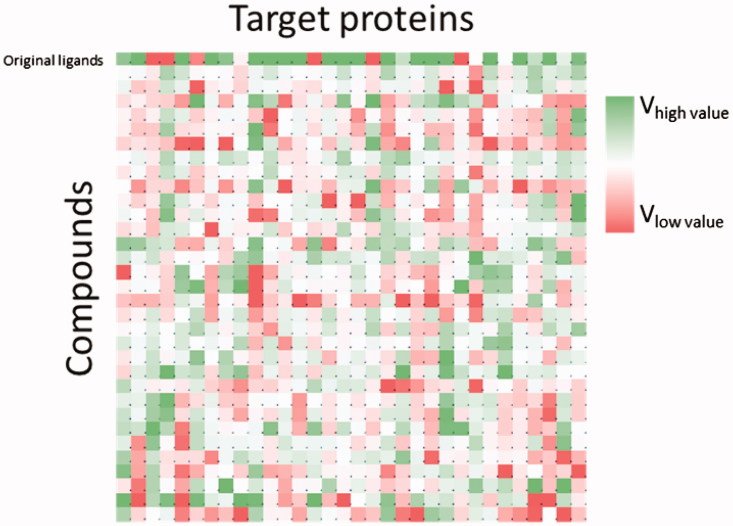
Matrix of results for calculated *V* values from the iVS analysis.

In this formula, *V* is the new value associated with each compound, *V*_0_ is the value of binding energy obtained from the docking calculation, *M*_L_ is the average binding energy of each ligand (in the different targets) and *M*_R_ is the average binding energy associated with each target (for the different ligands). Each single value in the matrix (Figure 1) represents the interaction between a single ligand *versus* a specific cellular protein (Table 3S and Figure1S–32S, Supporting Information). This was normalised by simultaneously taking into account the influence of the two specific averages from Equation (2). The values obtained led to the selection of various compounds against the different proteins, highlighting nine targets from the entire collection (i.e. PDB code: 3l3l, 3oyw, 4qmz, 2fb8, 3lbz, 4ks8, 4u5j, 4ual and 5h2u; for correspondence between PDB codes and proteins, see Table 1S, Supporting information). Specifically, these cellular proteins showed a higher trend of *V* values for the compound dataset, in comparison to the *V* values of the specific co-crystallised inhibitor. *V* values against the selected targets are summarised in Table 2.

**Table 3.** Selected hit compounds for each target.

Protein (PDB code)	Compounds								
3l3l	**2h**	**3a**	**3b**	**3c**	**3d**	**4g**	**6b**	**6c**	**6d**
3oyw	**1b**	**1e**	**2a**	**2b**	**2f**	**3a**	**3b**	**4g**	**6d**
4qmz	**1f**	**3c**	**3d**	**4b**	**4d**	**4f**	**4g**	**6c**	**6d**
2fb8	**2f**	**2g**	**4a**	**4c**	**4d**	**4e**	**4g**	**6a**	**6d**
3lbz	**2a**	**2h**	**2g**	**3c**	**4f**	**4h**	**5**	**6b**	**6d**
4ks8	**1b**	**1d**	**1f**	**2a**	**2b**	**2c**	**2h**	**4a**	**4b**
4u5j	**1f**	**2c**	**2h**	**3a**	**3c**	**4b**	**4d**	**4g**	**4h**
4ual	**1f**	**2g**	**3a**	**3c**	**3d**	**4c**	**6a**	**6c**	**6d**
5h2u	**4a**	**4b**	**4c**	**4d**	**4e**	**4f**	**4g**	**6c**	**6d**

Once identified the suitable targets for the library, we focussed on defining potency and overall binding affinity of the compounds. We used a cut-off of 30% potency to define the most active compounds for each protein. Interestingly, 27 out of 32 analogues demonstrated to possess high binding energies for one or more of the nine identified targets (i.e. 3l3l, 3oyw, 4qmz, 2fb8, 3lbz, 4ks8, 4u5j, 4ual and 5h2u). Indeed, some active compounds show high predicted affinity for more than one target, particularly compound **6d**. The lack of selectivity is not always desirable in drug discovery, although this behaviour could also represent an advantage (e.g. in the case of improved pharmacological effects of multi-target drugs)^35^ ^,^ ^36^. Therefore, additional mathematical filters (i.e. ligand efficiency or binding efficiency index) can be adopted for a more accurate analysis of the calculated selectivity for each compound. In contrast, five compounds were completely devoid of activity (i.e. **1a**,**c**, **2d,e** and **4j**), with regards to the calculated binding energies. Table 3 resumes the most potent ligands for each cellular protein.

As previously mentioned, the proteins analysed in this study play critical roles in tumour events and new binders are regarded as the potential agents for anti-cancer therapies^37–49^. Therefore, we examined literature records in order to cross-validate our method. Noteworthy, analogues based on the same *core* scaffolds have already demonstrated a good profile as inhibitors of the cellular targets analysed in this study. For instance, it has been reported that indole-based compounds are inhibitors of serine/threonine-protein kinase B-raf (BRAF)^50^ ^,^ ^51^, B-cell lymphoma 6 (BCL-6)^52^, proto-oncogene tyrosine-protein kinase Src (c-Src)^53^ ^,^ ^54^ and poly (ADP-ribose) polymerase (PARP)^55^, in clear agreement with our model. Similarly, indazole-based derivatives have been reported as inhibitors of BRAF^56^, phthalazinone *core* is present in inhibitors of PARP^57^, as well as quinoline scaffold is common in molecules acting as c-Src^58^ and mammalian sterile20-like protein kinase 3 (MST3) inhibitors^59^. These evidences validate our iVS method in order to enable the identification of suitable targets for a particular molecular library, foreseeing successful biological investigation.

### 2.3. Drug score and ADME assessment

The *in silico* assessment has been expanded through the evaluation of pharmacokinetic profiles and possible adverse side effects for the 32 new compounds reported in this study. In the first instance, we have determined the toxicity risk, the fragment-based druglikeness and the drug score (see Table 4S, Supporting Information; data calculated with DataWarrior version 4.7.2)^60^. The assessment of toxicity risk aims to locate substructures within the chemical structure which are indicative of risk within the four major toxicity classes – i.e. mutagenicity, tumorigenicity, irritating effects, and reproductive effects. The fragment-based druglikeness is based on a list of distinct substructure fragments with associated scores. The druglikeness is calculated summing up score values of those fragments that are present in the particular molecule under investigation. The drug score combines druglikeness, cLogP, LogS, molecular weight, and toxicity risks in one value that may be used to judge the overall potential of the compound to qualify as a drug. The results of these calculations for the entire library propose several compounds with a positive druglikeness and drug score > 0.3. In particular, **2 g**, **2 h**, **4 h**, **5** and **6d** demonstrated valuable profiles as drug candidate. For the five hits, the ADME properties were also calculated and the results are reported in Table 4. The results show that compounds **2 g**, **2 h**, **4 h**, **5** and **6d** exhibit also a good oral bioavailability (i.e. human intestinal absorption > 95%) and Caco-2 cell permeability >22 nm s^−1^ (Table 4). Although their high plasma protein binding (PPB >85%), **2 h**, **4 h**, **5** and **6d** are also supposed to satisfyingly permeate the blood-brain barrier (BBB penetration <1).

**Table 4.** *In silico* ADME profile of selected hits.

	Absorption^a^		Distribution^a^	
Compound	HIA (%)^b^	*In vitro* Caco-2 cell permeability (nm s^–1^)	*In vitro* PPB (%)	*In vivo* BBB penetration (C_brain_/C_blood_)
**2g**	96.96	51.63	91.53	1.82
**2h**	97.50	57.31	92.64	0.79
**4h**	96.36	25.24	86.88	0.06
**5**	96.16	22.38	90.14	0.56
**6d**	97.57	29.13	99.98	0.70

a The properties related to ADME were predicted using PreADMET web-based application (http://preadmet.bmdrc.kr).

b Human intestinal absorption (HIA, %).

## 3. Conclusions

We have described here the computational evaluation of a newly synthesised series of 32 heterocyclic small-molecules to explore molecular diversity and scaffold hopping through iVS approaches. Standard synthetic procedures allow the ease production of these compounds which are based on different heterocyclic scaffolds (i.e. indole, indazole, quinoline, naphtyridone, phthalazinone, and phthalhydrazide). The increase of scaffold diversity in small-molecules is recognised as an efficient way to implement the structural variation of molecular libraries, in order to reach specific interaction with a particular biological macromolecule.

iVS represents a validated computational tool for the assessment of binding towards targets of pharmacological interest. We have used this approach to define preliminary evaluation of the compound dataset *versus* a panel of cellular proteins involved in cancer progression and cancer cell survival. In the calculations, the normalisation of predicted binding energies allows to identify effective interactions for the compounds with nine biological targets – i.e. PARP, MST3, BCL6, c-Src, B-Raf kinase, galectin-1, serine/threonine-protein kinase PAK 6, serine/threonine-protein kinase MRCK beta, and protein-tyrosine kinase 6. Moreover, this study highlights a defined set of biological targets relevant for each active compound, which will drive subsequent biological screening.

## Supplementary Material

SI_v5.docx
