# Supplementary material for: iVS analysis to evaluate the impact of scaffold diversity in the binding to cellular targets relevant in cancer
Source: J Enzyme Inhib Med Chem. 2018 Oct 26;34(1):44–50. doi: 10.1080/14756366.2018.1518960 (PMC6211261; doi:10.1080/14756366.2018.1518960)
Supplement: SI_v5.docx [file IENZ_A_1518960_SM6536.docx]

**Supporting Information**

for

**iVS analysis to evaluate the impact of scaffold diversity in the binding to cellular targets relevant in cancer**

A. Cilibrizzi,^a,b,^* G. Floresta,^a,c^ V. Abbate^b^ and M. P. Giovannoni^d^

*^a^Institute of Pharmaceutical Science, King’s College London, Stamford Street, London SE1 9NH, UK*

*^b^King’s Forensics, School of Population Health & Environmental Sciences, King’s College London, Franklin-Wilkins Building, 150 Stamford Street, London SE1 9NH*

*^c^Department of Drug Sciences, University of Catania, V.le A. Doria, 95125 Catania, Italy*

*^d^NEUROFARBA, Sezione di Farmaceutica e Nutraceutica, Università degli Studi di Firenze, Via Ugo Schiff 6, 50019 Sesto Fiorentino, Italy*

**Content:**

1. Chemistry 3

1.1. Synthesis of indazole and indole derivatives 3

1.2. Synthesis of quinoline derivatives 4

1.3. Synthesis of naphtyridone derivatives 5

1.4. Synthesis of phthalazinone derivative 6

1.5. Synthesis of phthalhydrazide derivatives 7

2. Experimental Details - Chemistry 7

3. Experimental Details - Molecular modelling 21

4. References 42

**Tables:**

Table 1S. Selected protein targets for the iVS 22

Table 2S. ΔG results of the iVS calculations 23

Table 3S. Results of calculated V values (using eq. 2) 24

Table 4S. Results of the toxicity risk assessment and the fragment based druglikeness 41

**Captions:**

Figures 1S–32S. V values of co-crystallised ligands and screened compounds for each protein target 25-40

**1. Chemistry**

**1.1. Synthesis of indole and indazole derivatives**

Indazoles **1a-f** and indoles and **2a-f** were easily prepared in two steps using commercially available indazole-3-carboxylic acid **7a** and indole-3-carboxylic acid **7b** as starting materials (Scheme 1S). These were firstly treated with SOCl_2_ in the presence of Et_3_N to afford the intermediate acid chlorides. The addition of 4-butoxyaniline generated the corresponding amides **8a,b**, which were in turn transformed into the final compounds **1a,b** and **2a,b** through coupling with the appropriate phenylboronic acid in the presence of Cu(OAc)_2_. Differently, compounds **1c-f** and **2c-f** were prepared by standard alkylation of **8a,b** with suitable benzyl halides (Scheme 1S).

**Scheme 1S. Reagents and conditions: i)** SOCl_2_ (27 equiv), Et_3_N (catalytic), 1 h, 60 °C, then 4-*n.*butoxyaniline (2 equiv), anhydrous THF, 12 h, rt; **ii)** 3- or 4-methoxy-phenylboronic acid (2 equiv), Cu(OAc)_2_ (1.5 equiv), Et_3_N (2 equiv), CH_2_Cl_2_, 5-12 h, rt; **iii)** substituted benzyl halide (1.1 equiv), K_2_CO_3_ (2 equiv), anhydrous acetone, 2-10 h, reflux.

Schemes 2S and 3S show the synthetic pathways to prepare two “indomethacin-like” analogues. The amide intermediate **9** was obtained by coupling of 3-indoleacetic acid with 4-bromoaniline as previously described,^1^ using N,N'-Dicyclohexylcarbodiimide (DCC) to activate the carboxylic group. An alkylation reaction with 3-methoxybenyl chloride was performed to obtain the final compound **2g** (Scheme 2S). The same coupling reaction with 4-bromoaniline was also carried out on indomethacin **10**, to give the final compound **2h** (Scheme 3S).

**Scheme 2S. Reagents and conditions: i)** 3-methoxybenzyl chloride (1.5 equiv), K_2_CO_3_ (2 equiv), anhydrous acetone, 4 h, reflux.

**Scheme 3S. Reagents and conditions: i)** 4-bromoaniline (1 equiv), DCC (1 equiv), anhydrous CH_2_Cl_2_, 24 h, rt.

**1.2. Synthesis of quinoline derivatives**

Quinoline-based compounds **3a-d** were prepared in two steps (Scheme 4S). Commercially available 3-aminoquinoline **11** was firstly alkylated with the suitable benzyl halide in standard conditions, to give intermediates **12a-d**. These afforded the desired urea derivatives **3a-d** by treatment with 4-*n*.butoxyphenyl isocyanate.

**Scheme 4S. Reagents and conditions: i)** substituted benzyl halide (2 equiv), K_2_CO_3_ (2 equiv), anhydrous acetone, 3-7 h, 60 °C; **ii)** 4-*n*.butoxyphenyl isocyanate (1.1 equiv), anhydrous CH_2_Cl_2_, 3-8 h, rt.

**1.3. Synthesis of** **naphtyridone derivatives**

The naphtyridone scaffold **16** (Scheme 5S) was synthesised from commercially available 2-amino-6-methylpyridine **13** and diethyl-2-(ethoxymethylene)malonate **14**, through a following thermal ring closure of the diethyl-(6-methyl-2-pyridylaminomethylene)malonate intermediate **15**.^2,3^ **16** was then coupled with the appropriate phenylboronic acid in the presence of Cu(OAc)_2_ and an alkaline hydrolysis of the ester group afforded the carboxylic acids **18a,b**. These were firstly converted in the corresponding acid chlorides **19a,b** using SOCl_2_ at room temperature. 4-butoxyaniline was reacted with intermediates **19a,b** to give the final compounds **4a,b**. In contrast, the treatment of the acids **18a,b** with SOCl_2_ at 60 °C afforded the corresponding trichloromethyl acid chlorides **20a,b**. According to literature records, 2-methylpyridines, 2-methylquinolines and 2-methyl-4(1*H*)-quinolones react with different chlorinating agents to give the corresponding trichloromethyl derivatives.^4-7^ Lastly, **20a,b**, were transformed into the final products **4c,d** (Scheme 5S).

**Scheme 5S. Reagents and conditions: i)** 6 h, 100 °C; **ii)** diphenyl ether, 0.5 h, 270 °C; **iii)** 3- or 4-methoxyphenylboronic acid (2 equiv), Cu(OAc)_2_ (1.5 equiv), Et_3_N (2 equiv), CH_2_Cl_2_, 15-20 h, rt; **iv)** NaOH 6N, EtOH, 0.5-1 h, rt; **v)** SOCl_2_ (27 equiv), Et_3_N (catalytic), 2-3 h, rt; **vi)** 4-*n.*butoxyaniline (2 equiv), anhydrous THF, 2 h, rt; **vii)** SOCl_2_ (27 equiv), Et_3_N (catalytic), 2-3 h, 60 °C; **viii)** substituted benzyl halide (1.1 equiv), K_2_CO_3_ (2 equiv), anhydrous acetone, 5-7 h, 60 °C; **ix)** 4-*n.*butoxyaniline (1 equiv), DCP (4 equiv), Et_3_N (catalytic), anhydrous DMF, 15 h, rt.

The synthesis of analogues **4e-g** (Scheme 5S) was performed in standard condition starting from intermediate **16**, through alkylation (**21a-c**) and alkaline hydrolysis, to give the carboxylic acids **22a-c**. In the last step, **22a-c** were treated with 4-*n*.butoxyaniline, using a catalytic amount of Et_3_N and diethyl cyanophosphonate (DCP) to activate the carboxylic group (Scheme 5S), in order to produce the final compounds **4e-g**.

Scheme 6S shows the synthetic pathway to prepare two nalidixic acid-based derivatives. The treatment of nalidixic acid **23** with ethyl chloroformate afforded the intermediate mixed anhydride, which was transformed into the final amide **4h**. Differently, compound **4j** was obtained through the same conditions reported above for the trichloromethyl analogues **4c,d** (Scheme 5S).

**Scheme 6S. Reagents and conditions: i)** ethyl chloroformate (1.1 equiv), Et_3_N (3.5 equiv), 4-bromoaniline (2 equiv), anhydrous THF, 12 h, -5 °C → rt; **ii)** SOCl_2_ (27 equiv), Et_3_N (catalytic), 2 h, 60 °C, then 4-*n*.butoxyaniline (2 equiv), anhydrous THF, 2 h, rt.

**1.4. Synthesis of phthalazinone derivative**

The synthetic route employed to obtain the phthalazinone analogue **5** is depicted in Scheme 7S. 4-Bromoaniline was firstly converted into the phenylamide intermediate **24**,^8^ as previously described. Subsequently, alkylation in standard conditions of the phthalazin-1(2*H*)-one with **24** afforded the final compound **5**.

**Scheme 7S. Reagents and conditions: i)** 1-(2*H*)**-**phthalazinone (0.9 equiv), K_2_CO_3_ (1.8 equiv), anhydrous CH_3_CN, 3 h, reflux.

**1.5. Synthesis of** **phthalhydrazide** **derivatives**

Scheme 8S shows the synthetic procedure for final compounds **6a-d**. The first step is the alkylation in standard conditions of commercially available 2,3-diidrophthalazin-1,4-dione **25** with a suitable benzyl halide. For this reaction, anhydrous DMF demonstrated the best solvent in terms of mono- *vs.* bi-alkylated ratio, although traces of product alkylated on the two nitrogen atoms were detected in all the attempts. The previously described intermediate **26c**^9^ and the new **26a,b** were converted in the final urea derivatives **6a-c** using 4-*n*-butoxyphenyl isocyanate in anhydrous CH_2_Cl_2_. Differently, the analogue **6d** was synthesised through standard alkylation of **26b** with intermediate **24** (Scheme 7S).

**Scheme 8S. Reagents and conditions: i)** substituted benzyl halide (1.1 equiv), K_2_CO_3_ (2 equiv), anhydrous DMF, 2-4 h, 80 °C; **ii)** 4-*n.*butoxyphenyl isocyanate (2 equiv), anhydrous CH_2_Cl_2_, 14 h, 0 °C → rt; **iii)** **24** (1.5 equiv), K_2_CO_3_ (2 equiv), anhydrous CH_3_CN, 3 h, reflux.

**2.** **Experimental Details - Chemistry**

**2.1. General remarks**

Reagents and starting materials were obtained from commercial sources. Extracts were dried over Na_2_SO_4_, and the solvents were removed under reduced pressure. All reactions were monitored by thin layer chromatography (TLC) using commercial plates pre-coated with Merck silica gel 60 F-254. Visualisation was performed by UV fluorescence (λ_max_ = 254 nm) or by staining with iodine or potassium permanganate. Chromatographic separations were performed on a silica gel column by gravity (Kieselgel 40, 0.063-0.200 mm; Merck) or flash chromatography (Kieselgel 40, 0.040-0.063 mm; Merck). Yields refer to chromatographically and spectroscopically pure compounds, unless otherwise stated. When reactions were performed in anhydrous conditions, the mixtures were maintained under nitrogen atmosphere. Compounds were named following IUPAC rules as applied by Beilstein-Institut AutoNom 2000 (4.01.305) or CA Index Name. The identity and purity of intermediates and final compounds was ascertained through NMR and TLC chromatography. All melting points were determined on a microscope hot stage Büchi apparatus and are uncorrected. Mass spectra (m/z) were recorded on ESI-TOF mass spectrometer (Bruker Micro TOF) and reported mass values are within the error limits of ± 5 ppm mass units. ^1^H NMR spectra were recorded with Avance 400 instruments (Bruker Biospin Version 002 with SGU). Chemical shifts (*δ*) are reported in ppm to the nearest 0.01 ppm, using the solvent as internal standard. Coupling constants (*J* values) are given in Hz and were calculated using ‘TopSpin 1.3’ software rounded to the nearest 0.1 Hz. Data are reported as follows: chemical shift, multiplicity [exch, exchange; br, broad; s, singlet; d, doublet; t, triplet; q, quartet; quin, quintet; sext, sextet; sept, septet; m, multiplet; or as a combination of these (e.g. dd, dt *etc*.)], integration, assignment and coupling constant(s).

**2.1.1. General Procedure for 1a,b**

Et_3_N (0.2 mL) was added to a cooled (0 °C) and stirred suspension of commercially available indazole-3-carboxylic **7a** or indole-3-carboxylic acid **7b** (1.55 mmol) in SOCl_2_ (3 mL). After 1 h at 60 °C, the mixture was cooled and the excess of SOCl_2_ was removed under vacuum. The residue was then dissolved in anhydrous THF (1.5 mL) and cooled at 0 °C. A solution of 4-*n.*butoxyaniline (3.10 mmol) in anhydrous THF (1 mL) was added dropwise and the reaction was carried out at room temperature for 12 h. The mixture was concentrated *in vacuo*, diluted with ice-cold water (10 mL) and kept under stirring at 0 °C for 0.5 h. The precipitate was recovered by filtration and purified by crystallisation from ethanol to afford intermediates **8a,b**.

***N*-(4-Butoxyphenyl)-1*H*-indazole-3-carboxamide (8a).** Yield = 97 %; mp = 157-159 °C (EtOH). ^1^H NMR (CDCl_3_) *δ* 1.01 (t, 3H, CH_2_*CH_3_*, *J* = 6.7 Hz), 1.52 (sext, 2H, *CH_2_*CH_3_, *J* = 7.4 Hz), 1.80 (quin, 2H, CH_2_*CH_2_*CH_2_, *J* = 6.9 Hz), 4.00 (t, 2H, OCH_2_, *J* = 6.6 Hz), 6.94 (d, 2H, Ar, *J* = 8.3 Hz), 7.36 (t, 1H, Ar, *J* = 8.0 Hz), 7.50 (t, 1H, Ar, *J* = 8.4 Hz), 7.57 (d, 1H, Ar, *J* = 8.4 Hz), 7.67 (d, 2H, Ar, *J* = 8.3 Hz), 8.49 (d, 1H, Ar, *J* = 8.2 Hz), 8.85 (exch br s, 1H, NH), 10.42 (exch br s, 1H, NH).

***N*-(4-Butoxyphenyl)-1*H*-indole-3-carboxamide (8b).** Yield = 94 %; mp = 181-182 °C (EtOH). ^1^H NMR (CDCl_3_) *δ* 1.01 (t, 3H, CH_2_*CH_3_*, *J* = 7.3 Hz), 1.52 (sext, 2H, *CH_2_*CH_3_, *J* = 7.5 Hz), 1.79 (quin, 2H, CH_2_*CH_2_*CH_2_, *J* = 6.7 Hz), 3.97 (t, 2H, OCH_2_, *J* = 6.4 Hz), 6.93 (d, 2H, Ar, *J* = 8.4 Hz), 7.31 (t, 2H, Ar, *J* = 3.2 Hz), 7.48 (d, 1H, Ar, *J* = 5.9 Hz), 7.56 (d, 2H, Ar, *J* = 8.5 Hz), 7.86 (s, 1H, Ar), 8.06 (d, 1H, Ar, *J* = 3.7 Hz), 8.78 (exch br s, 1H, NH), 9.21 (exch br s, 1H, NH).

**2.1.2. General Procedure for 1a,b and 2a,b.**

Et_3_N (0.96 mmol) was added to a suspension of **8a** or **8b** (0.32 mmol), copper acetate (0.48 mmol) and 3- or 4-methoxyphenylboronic acid (0.64 mmol) in CH_2_Cl_2_ (2 mL). The mixture was stirred at room temperature for 5-12 h, diluted with additional CH_2_Cl_2_ (8 mL) and extracted with 15% aqueous ammonia (10 mL). The organic layer was washed with 10 mL of water and dried over Na_2_SO_4_. After removal of the solvent, the residue was purified by flash column chromatography using CH_2_Cl_2_ (for **1a,b**) and CH_2_Cl_2_/MeOH 99:1 (for **2a,b**) as eluents.

***N*-(4-Butoxyphenyl)-1-(3-methoxyphenyl)-1*H*-indazole-3-carboxamide (1a).** Yield = 46 %; yellowish oil. ^1^H NMR (CDCl_3_) *δ* 1.01 (t, 3H, CH_2_*CH_3_*, *J* = 7.4 Hz), 1.53 (sext, 2H, *CH_2_*CH_3_, *J* = 7.4 Hz), 1.80 (quin, 2H, CH_2_*CH_2_*CH_2_, *J* = 8.1 Hz), 3.94 (s, 3H, OCH_3_), 4.00 (t, 2H, OCH_2_, *J* = 6.5 Hz), 6.95 (d, 2H, Ar, *J* = 7.9 Hz), 7.03 (dd, 1H, Ar, *J* = 6.1 Hz, *J* = 2.2 Hz), 7.36 (s, 1H, Ar), 7.38 (dd, 1H, Ar, *J* = 1.0 Hz, *J* = 1.0 Hz), 7.42 (d, 1H, Ar, *J* = 7.9 Hz) 7.52 (t, 2H, Ar, *J* = 8.2 Hz), 7.68 (d, 2H, Ar, *J* = 8.9 Hz), 7.77 (d, 1H, Ar, *J* = 8.6 Hz), 8.56 (d, 1H, Ar, *J* = 8.1 Hz), 8.86 (exch br s, 1H, NH).

***N*-(4-Butoxyphenyl)-1-(4-methoxyphenyl)-1*H*-indazole-3-carboxamide (1b).** Yield = 60 %; yellowish oil. ^1^H NMR (CDCl_3_) *δ* 1.01 (t, 3H, CH_2_*CH_3_*, *J* = 7.5 Hz), 1.53 (sext, 2H, *CH_2_*CH_3_, *J* = 7.4 Hz), 1.80 (quin, 2H, CH_2_*CH_2_*CH_2_, *J* = 8.0 Hz), 3.94 (s, 3H, OCH_3_), 4.00 (t, 2H, OCH_2_, *J* = 6.6 Hz), 6.94 (d, 2H, Ar, *J* = 8.8 Hz), 7.13 (d, 2H, Ar, *J* = 9.0 Hz), 7.39 (t, 1H, Ar, *J* = 7.0 Hz), 7.49 (t, 1H, Ar, *J* = 8.4 Hz), 7.63-7.70 (m, 5H, Ar), 8.55 (d, 1H, Ar, *J* = 8.1 Hz), 8.85 (exch br s, 1H, NH).

***N*-(4-Butoxyphenyl)-1-(3-methoxyphenyl)-1*H*-indole-3-carboxamide (2a).** Yield = 23 %; yellowish oil. ^1^H NMR (CDCl_3_) *δ* 1.01 (t, 3H, CH_2_*CH_3_*, *J* = 7.4 Hz), 1.53 (sext, 2H, *CH_2_*CH_3_, *J* = 7.4 Hz), 1.80 (quin, 2H, CH_2_*CH_2_*CH_2_, *J* = 7.8 Hz), 3.90 (s, 3H, OCH_3_), 4.00 (t, 2H, OCH_2_, *J* = 6.6 Hz), 6.94 (d, 2H, Ar, *J* = 6.5 Hz), 7.01 (d, 1H, Ar, *J* = 8.2 Hz), 7.06 (s, 1H, Ar), 7.12 (d, 1H, Ar, *J* = 7.4 Hz), 7.36 (quin, 2H, Ar, *J* = 7.3 Hz), 7.48 (t, 1H, Ar, *J* = 8.0 Hz), 7.56-1.62 (m, 3H, Ar), 7.94 (br s, 1H, Ar), 8.15 (d, 2H, Ar, *J* = 7.2 Hz), 9.72 (exch br s, 1H, NH).

***N*-(4-Butoxyphenyl)-1-(4-methoxyphenyl)-1*H*-indole-3-carboxamide (2b).** Yield = 15 %; yellowish oil. ^1^H NMR (CDCl_3_) *δ* 1.01 (t, 3H, CH_2_*CH_3_*, *J* = 7.4 Hz), 1.48 (sext, 2H, *CH_2_*CH_3_, *J* = 7.4 Hz), 1.80 (quin, 2H, CH_2_*CH_2_*CH_2_, *J* = 7.8 Hz), 3.92 (s, 3H, OCH_3_), 4.00 (t, 2H, OCH_2_, *J* = 6.5 Hz), 6.94 (d, 2H, Ar, *J* = 8.9 Hz), 7.08 (d, 2H, Ar, *J* = 8.8 Hz), 7.33 (quin, 2H, Ar, *J* = 8.1 Hz), 7.42 (d, 2H, Ar, *J* = 8.7 Hz), 7.47 (d, 1H, Ar, *J* = 8.3 Hz), 7.57(d, 2H, Ar, *J* = 8.9 Hz ), 7.88 (br s, 1H, Ar), 8.14 (d, 1H, Ar, *J* = 7.2 Hz).

**2.1.3. General Procedure for 1c-f and 2c-f.**

Compounds **1c-f** and **2c-f** were obtained starting from **8a** (for **1c-f**) and from **8b** (for **2c-f**), respectively. A mixture of **8a** or **8b** (0.32 mmol), K_2_CO_3_ (0.65 mmol) and the appropriate benzyl halide (0.36 mmol) was stirred in anhydrous acetone (2 mL) under reflux for 2-10 h. The mixture was then concentrated *in vacuo*, diluted with cold water and extracted with CH_2_Cl_2_ (3 x 15 mL). The organic layer was dried over Na_2_SO_4_ and evaporated *in vacuo*. Compounds **1c-f** and **2c-f** were purified by column chromatography using CH_2_Cl_2_ (for **1c-f** and **2e**) or CH_2_Cl_2_/MeOH 99:1 (for **2c,d,f**) as eluents.

***N*-(4-Butoxyphenyl)-1-(3-methoxybenzyl)-1*H*-indazole-3-carboxamide (1c).** Yield = 65 %; yellowish oil. ^1^H NMR (CDCl_3_) *δ* 1.00 (t, 3H, CH_2_*CH_3_*, *J* = 7.4 Hz), 1.52 (sext, 2H, *CH_2_*CH_3_, *J* = 5.4 Hz), 1.79 (quin, 2H, CH_2_*CH_2_*CH_2_, *J* = 6.4 Hz), 3.63 (s, 3H, OCH_3_), 4.00 (t, 2H, OCH_2_, *J* = 6.5 Hz), 5.74 (s, 2H, CH_2_N), 6.29 (s, 1H, Ar), 6.80 (d, 1H, Ar, *J* = 8.8 Hz), 6.94 (d, 2H, Ar, *J* = 9.0 Hz), 7.29 (s, 1H, Ar), 7.36 (d, 2H, Ar, *J* = 8.8 Hz), 7.44 (t, 2H, Ar, *J* = 8.2 Hz), 7.66 (d, 2H, Ar, *J* = 9.0 Hz), 8.49 (d, 1H, Ar, *J* = 8.2 Hz), 8.77 (exch br s, 1H, NH).

***N*-(4-Butoxyphenyl)-1-(4-methoxybenzyl)-1*H*-indazole-3-carboxamide (1d).** Yield = 73 %; mp = 115-16 °C (EtOH). ^1^H NMR (CDCl_3_) *δ* 1.01 (t, 3H, CH_2_*CH_3_*, *J* = 7.4 Hz), 1.53 (sext, 2H, *CH_2_*CH_3_, *J* = 7.4 Hz), 1.78 (quin, 2H, CH_2_*CH_2_*CH_2_, *J* = 8.0 Hz), 3.79 (s, 3H, OCH_3_), 3.99 (t, 2H, OCH_2_, *J* = 6.5 Hz), 5.60 (s, 2H, CH_2_N), 6.87 (d, 2H, Ar, *J* = 8.5 Hz), 6.94 (d, 2H, Ar, *J* = 8.9 Hz), 7.19 (d, 2H, Ar, *J* = 8.6 Hz), 7.30-7.34 (m, 1H, Ar), 7.40 (s, 2H, Ar), 7.67 (d, 2H, Ar, *J* = 8.9 Hz), 8.47 (d, 1H, Ar, *J* = 8.2 Hz), 8.80 (exch br s, 1H, NH).

***N*-(4-Butoxyphenyl)-1-(3-chlorobenzyl)-1*H*-indazole-3-carboxamide (1e).** Yield = 86 %; mp = 119-20 °C (EtOH). ^1^H NMR (CDCl_3_) *δ* 1.01 (t, 3H, CH_2_*CH_3_*, *J* = 7.4 Hz), 1.52 (sext, 2H, *CH_2_*CH_3_, *J* = 7.6 Hz), 1.81 (quin, 2H, CH_2_*CH_2_*CH_2_, *J* = 6.6 Hz), 4.00 (t, 2H, OCH_2_, *J* = 6.5 Hz), 5.63 (s, 2H, CH_2_N), 6.94 (d, 2H, Ar, *J* = 9.0 Hz), 7.08 (d, 2H, Ar, *J* = 6.7 Hz), 7.24 (s, 1H, Ar), 7.30-7.27 (m, 1H, Ar), 7.33-7.39 (m, 2H, Ar), 7.43-7.47 (m, 1H, Ar), 7.67 (d, 2H, Ar, *J* = 9.0 Hz), 8.49 (d, 1H, Ar, *J* = 8.1 Hz), 8.76 (exch br s, 1H, NH).

**1-(1,3-Benzodioxol-5-ylmethyl)-*N*-(4-butoxyphenyl)-1*H*-indazole-3-carboxamide (1f).** Yield = 78 %; mp = 134-35 °C (EtOH). ^1^H NMR (CDCl_3_) *δ* 1.01 (t, 3H, CH_2_*CH_3_*, *J* = 7.3 Hz), 1.53 (sext, 2H, *CH_2_*CH_3_, *J* = 7.4 Hz), 1.76-1.83 (m, 2H, CH_2_*CH_2_*CH_2_), 4.00 (t, 2H, OCH_2_, *J* = 6.5 Hz), 5.56 (s, 2H, CH_2_N), 5.95 (s, 2H, OCH_2_O), 6.72 (s, 1H, Ar), 6.78 (s, 2H, Ar), 6.94 (d, 2H, Ar, *J* = 8.9 Hz), 7.30-7.34 (m, 1H, Ar), 7.42 (s, 2H, Ar), 7.67 (d, 2H, Ar, *J* = 8.9 Hz), 8.47 (d, 1H, Ar, *J* = 8.1 Hz), 8.78 (exch br s, 1H, NH).

***N*-(4-Butoxyphenyl)-1-(3-methoxybenzyl)-1*H*-indole-3-carboxamide (2c).** Yield = 58 %; mp = 166-67 °C (EtOH). ^1^H NMR (CDCl_3_) *δ* 1.01 (t, 3H, CH_2_*CH_3_*, *J* = 7.3 Hz), 1.52 (sext, 2H, *CH_2_*CH_3_, *J* = 7.6 Hz), 1.80 (quin, 2H, CH_2_*CH_2_*CH_2_, *J* = 7.0 Hz), 3.77 (s, 3H, OCH_3_), 3.99 (t, 2H, OCH_2_, *J* = 6.5 Hz), 5.31 (s, 2H, CH_2_N), 6.71 (s, 1H, Ar), 6.75 (d, 1H, Ar, *J* = 7.5 Hz), 6.85 (d, 1H, Ar, *J* = 8.3 Hz), 6.92 (d, 2H, Ar, *J* = 8.8 Hz) 7.25 (d, 1H, Ar, *J* = 7.8 Hz), 7.29-7.33 (m, 2H, Ar), 7.38 (d, 1H, Ar, *J* = 8.0 Hz), 7.53 (d, 2H, Ar, *J* = 8.3 Hz), 7.73 (br s, 1H, Ar), 8.10 (d, 1H, Ar, *J* = 7.1 Hz).

***N*-(4-Butoxyphenyl)-1-(4-methoxybenzyl)-1*H*-indole-3-carboxamide (2d).** Yield = 29 %; mp = 160-62 °C (EtOH). ^1^H NMR (CDCl_3_) *δ* 1.01 (t, 3H, CH_2_*CH_3_*, *J* = 7.4 Hz), 1.52 (sext, 2H, *CH_2_*CH_3_, *J* = 7.6 Hz), 1.79 (quin, 2H, CH_2_*CH_2_*CH_2_, *J* = 7.1 Hz), 3.81 (s, 3H, OCH_3_), 3.98 (t, 2H, OCH_2_, *J* = 6.6 Hz), 5.28 (s, 2H, CH_2_N), 6.89 (q, 4H, Ar, *J* = 8.4 Hz), 7.13 (d, 2H, Ar, *J* = 8.7 Hz), 7.29-7.34 (m, 2H, Ar), 7.40 (d, 1H, Ar, *J* = 8.8 Hz), 7.53 (d, 2H, Ar, *J* = 8.9 Hz), 7.73 (br s, 1H, Ar), 8.10 (d, 1H, Ar, *J* = 12.1 Hz).

***N*-(4-Butoxyphenyl)-1-(3-chlorobenzyl)-1*H*-indole-3-carboxamide (2e).** Yield = 51 %; mp = 136-37 °C (EtOH). ^1^H NMR (CDCl_3_) *δ* 1.01 (t, 3H, CH_2_*CH_3_*, *J* = 7.3 Hz), 1.52 (sext, 2H, *CH_2_*CH_3_, *J* = 7.6 Hz), 1.80 (quin, 2H, CH_2_*CH_2_*CH_2_, *J* = 7.0 Hz), 3.99 (t, 2H, OCH_2_, *J* = 6.5 Hz), 5.32 (s, 2H, CH_2_N), 6.92 (d, 2H, Ar, *J* = 8.9 Hz), 7.02 (d, 1H, Ar, *J* = 7.1 Hz), 7.17 (s, 1H, Ar), 7.25-7.34 (m, 5H, Ar), 7.53 (d, 2H, Ar, *J* = 8.3 Hz), 7.75 (br s, 1H, Ar), 8.10 (d, 1H, Ar, *J* = 8.4 Hz).

**1-(1,3-Benzodioxol-5-ylmethyl)-*N*-(4-butoxyphenyl)-1*H*-indole-3-carboxamide (2f).** Yield = 42 %; mp = 165-67 °C (EtOH). ^1^H NMR (CDCl_3_) *δ* 1.01 (t, 3H, CH_2_*CH_3_*, *J* = 7.4 Hz), 1.52 (sext, 2H, *CH_2_*CH_3_, *J* = 7.4 Hz), 1.80 (quin, 2H, CH_2_*CH_2_*CH_2_, *J* = 6.8 Hz), 3.99 (t, 2H, OCH_2_, *J* = 6.6 Hz), 5.23 (s, 2H, CH_2_N), 5.96 (s, 2H, OCH_2_O), 6.64 (s, 1H, Ar, *J* = 8.4 Hz), 6.71 (d, 1H, Ar, *J* = 7.9 Hz), 6.78 (d, 1H, Ar, *J* = 7.9 Hz), 6.92 (d, 2H, Ar, J = 8.9 Hz), 7.31-7.33 (m, 2H, Ar), 7.40 (m, 1H, Ar), 7.53 (d, 2H, Ar, *J* = 8.2 Hz), 7.71 (br s, 1H, Ar), 8.09 (d, 1H, Ar, *J* = 7.7 Hz).

**2.1.4. *N*-(4-Bromophenyl)-2-(1-(3-methoxybenzyl)-1*H*-indol-3-yl)acetamide (2g).**

A mixture of **9** (0.46 mmol), K_2_CO_3_ (0.92 mmol) and 3-methoxybenzyl chloride (0.69 mmol) was refluxed in anhydrous acetone (3 mL) under stirring for 4 h. After removal of the solvent, the residue was suspended in ice-cold water and extracted with CH_2_Cl_2_ (3 x 15 mL). The organic solvent was evaporated *in vacuo* to afford the crude **2g**, which was purified by column chromatography using toluene/ethyl acetate 7:3 as eluent. Yield = 29 %; mp = 153-55 °C (EtOH). ^1^H NMR (CDCl_3_) *δ* 3,77 (s, 3H, OCH_3_); 3,90 (s, 2H, COCH_2_); 5,33 (s, 2H, CH_2_N); 6,68 (s, 1H, CH); 6,76 (d, 1H, Ar, *J* = 7.1 Hz); 7,84 (dd, 1H, Ar, *J* = 8.5 Hz, *J* = 1.9 Hz ); 7,17-7,30 (m, 5H, Ar); 7,36-7,41 (m, 4H, Ar); 7,63 (d, 1H, Ar, *J* = 8.2 Hz).

**2.1.5. *N*-(4-Bromophenyl)-2-[1-(4-chlorobenzoyl)-5-methoxy-2-methyl-1*H*-indol-3-yl]acetamide (2h).**

DCC (0.28 mmol) and 4-bromoaniline (0.28 mmol) were added to a solution of indomethacin **10** (0.28 mmol) in anhydrous CH_2_Cl_2_ (5 ml). The reaction mixture was stirred at room temperature for 24 h. The precipitate was filtered off and the solution was washed with 2N HCl (2 x 10 mL) and with H_2_O (10 mL). The organic phase was dried over NaSO_4_ and concentrated under reduce pressure. The crude residue was purified by flash column chromatography using CH_2_Cl_2_/MeOH 99:1 as eluent to obtain **2h** as a white solid. Yield = 35 %; mp = 200-01 °C (EtOH). ^1^H NMR (CDCl_3_) *δ* 2,48 (s, 3H, CH_3_); 3,83 (s, 5H, OCH_3_ + COCH_2_); 6,74 (dd, 1H, Ar, *J* = 6.6 Hz, *J* = 2.5 Hz); 6,88 (d, 1H, Ar, *J* = 9.1 Hz); 6,94 (d, 1H, Ar, *J* = 2.3 Hz); 7,25 (exch, br, s, 1H, NH); 7,31 (d, 2H, Ar, *J* = 8.8 Hz); 7,41 (d, 2H, Ar, *J* = 8.7 Hz); 7,52 (d, 2H, Ar, *J* = 8.4 Hz); 7,71 (d, 2H, Ar, *J* = 8.4 Hz).

**2.1.6. General Procedure for 12a-d.**

A mixture of 3-aminoquinoline **11** (0.69 mmol), K_2_CO_3_ (1.38 mmol) and the appropriate benzyl halide (0.69 mmol) was refluxed in anhydrous acetone (3 mL) under stirring for 1-3 h. Additional benzyl halide (0.69 mmol) was added and the reaction was kept at reflux for further 2-4 h. The mixture was then concentrated under vacuum, diluted with cold water and extracted with CH_2_Cl_2_ (3 x 15 mL). The organic layer was dried over Na_2_SO_4_ and evaporated *in vacuo* to afford the crude compounds **12a-d** which were purified by column chromatography using cyclohexane/ethyl acetate 1:2 (for **12a,b,d**) or 2:1 (for **12c**) as eluents.

***N*-(3-Methoxybenzyl)quinolin-3-amine (12a).** Yield = 49 %; clear oil. ^1^H NMR (CDCl_3_) *δ* 3.82 (s, 3H, OCH_3_), 4.44 (s, 2H, CH_2_N), 4.67 (exch br s, 1H, NH), 6.86 (dd, 1H, Ar, *J* = 8.2 Hz, *J* = 2.5 Hz), 6.98 (s, 1H, Ar), 7.02 (d, 1H, Ar, *J* = 7.6 Hz), 7.07 (d, 1H, Ar, *J* = 2.7 Hz), 7.31 (t, 1H, Ar, *J* = 7.9 Hz), 7.43-7.45 (m, 2H, Ar), 7.59-7.61 (m, 2H, Ar), 7.98-8.01 (m, 1H, Ar), 8.59 (d, 1H, Ar, *J* = 2.8 Hz).

***N*-(4-Methoxybenzyl)quinolin-3-amine (12b).** Yield = 60 %; mp = 80-82 °C (EtOH). ^1^H NMR (CDCl_3_) *δ* 3.83 (s, 3H, OCH_3_), 4.37 (s, 2H, CH_2_N), 4.43 (exch br s, 1H, NH), 6.92 (d, 2H, Ar, *J* = 8.7 Hz), 7.05 (d, 1H, Ar, *J* = 2.7 Hz), 7.34 (d, 2H, Ar, *J* = 8.6 Hz), 7.42-7.44 (m, 2H, Ar), 7.59-7.63 (m, 1H, Ar), 7.95-7.98 (m, 1H, Ar), 8.50 (d, 1H, Ar, *J* = 2.8 Hz).

***N*-(1,3-Benzodioxol-5-ylmethyl)quinolin-3-amine (12c).** Yield = 26 %; brown oil. ^1^H NMR (CDCl_3_) *δ* 4.40 (s, 2H, CH_2_N), 5.21 (exch br s, 1H, NH), 5.97 (s, 3H, OCH_2_O), 6.81 (d, 1H, Ar, *J* = 7.8 Hz), 6.91 (d, 1H, Ar, *J* = 11.2 Hz), 7.13 (s, 1H, Ar), 7.46-7.49 (m, 2H, Ar), 7.62-7.65 (m, 1H, Ar), 8.06-8.09 (m, 1H, Ar), 8.78 (s, 1H, Ar).

***N*-(3-Chlorobenzyl)quinolin-3-amine (12d).** Yield = 22 %; clear oil. ^1^H NMR (CDCl_3_) *δ* 4.51 (d, 2H, CH_2_N, *J* = 4.5 Hz), 5.73 (exch br s, 1H, NH), 7.13 (d, 1H, Ar, *J* = 2.6 Hz), 7.29-7.34 (m, 3H, Ar), 7.44 (s, 1H, Ar), 7.49-7.52 (m, 2H, Ar), 7.63-7.65 (m, 1H, Ar), 8.13 (t, 1H, Ar, *J* = 6.3 Hz), 8.94 (s, 1H, Ar).

**2.1.7. General Procedure for 3a-d.**

The suitable amine **12a-d** (0.34 mmol) was dissolved in 2 mL of anhydrous CH_2_Cl_2_ and 4-*n*.butoxyphenyl isocyanate (0.37 mmol) was added under stirring. The reaction was carried out at room temperature for 3-8 h. The solid residue was filtered off and the solution was evaporated *in vacuo* to afford compounds **3a-d**, which were purified by flash column chromatography using cyclohexane/ethyl acetate 1:1 (for **3a,b**) or 2:1 (for **3c,d**) as eluents.

**3-(4-Butoxyphenyl)-1-(3-methoxybenzyl)-1-(quinolin-3-yl)urea (3a).** Yield = 58 %; clear oil. ^1^H NMR (CDCl_3_) *δ* 0.98 (t, 3H, CH_2_*CH_3_*, *J* = 7.4 Hz), 1.48 (sext, 2H, *CH_2_*CH_3_, *J* = 7.6 Hz), 1.75 (quin, 2H, CH_2_*CH_2_*CH_2_, *J* = 7.0 Hz), 3.77 (s, 3H, OCH_3_), 3.93 (t, 2H, OCH_2_, *J* = 6.5 Hz), 5.04 (s, 2H, CH_2_N), 6.10 (exch br s, 1H, NH), 6.81-6.84 (m, 3H, Ar), 6.88 (d, 2H, Ar, *J* = 7.4 Hz), 7.19-7.25 (m, 3H, Ar), 7.63 (t, 1H, Ar, *J* = 8.2 Hz), 7.78-7.82 (m, 2H, Ar), 8.01 (s, 1H, Ar), 8.18 (d, 1H, Ar, *J* = 8.7 Hz), 8.79 (d, 1H, Ar, *J* = 2.4 Hz).

**3-(4-Butoxyphenyl)-1-(4-methoxybenzyl)-1-(quinolin-3-yl)urea (3b).** Yield = 47 %; clear oil. ^1^H NMR (CDCl_3_) *δ* 0.97 (t, 3H, CH_2_*CH_3_*, *J* = 7.4 Hz), 1.48 (sext, 2H, *CH_2_*CH_3_, *J* = 7.6 Hz), 1.74 (quin, 2H, CH_2_*CH_2_*CH_2_, *J* = 7.0 Hz), 3.79 (s, 3H, OCH_3_), 3.92 (t, 2H, OCH_2_, *J* = 6.5 Hz), 4.97 (s, 2H, CH_2_N), 6.18 (exch br s, 1H, NH), 6.81 (dd, 4H, Ar, *J* = 2.0 Hz, *J* = 6.6 Hz), 7.21 (dd, 4H, Ar, *J* = 6.4 Hz, *J* = 2.1 Hz), 7.62 (t, 1H, Ar, *J* = 7.0 Hz), 7.78 (t, 2H, Ar, *J* = 7.2 Hz), 7.97 (s, 1H, Ar), 8.14 (d, 1H, Ar, *J* = 8.0 Hz), 8.71 (d, 1H, Ar, *J* = 2.3 Hz).

**1-(1,3-Benzodioxol-5-ylmethyl)-3-(4-butoxyphenyl)-1-(quinolin-3-yl)urea (3c).** Yield = 36 %; brown oil. ^1^H NMR (CDCl_3_) *δ* 0.97 (t, 3H, CH_2_*CH_3_*, *J* = 7.4 Hz), 1.48 (sext, 2H, *CH_2_*CH_3_, *J* = 7.6 Hz), 1.75 (quin, 2H, CH_2_*CH_2_*CH_2_, *J* = 7.0 Hz), 3.92 (t, 2H, OCH_2_, *J* = 6.6 Hz), 4.96 (s, 2H, CH_2_N), 5.96 (s, 3H, OCH_3_), 6.20 (exch br s, 1H, NH), 6.65-6-71 (m, 2H, Ar), 6.81 (d, 2H, Ar, *J* = 9.0 Hz), 6.91 (s, 1H, Ar), 7.21 (d, 2H, Ar, *J* = 9.0 Hz), 7.66 (t, 1H, Ar, *J* = 7.2 Hz), 7.82 (q, 2H, Ar, *J* = 6.7 Hz), 8.06 (s, 1H, Ar), 8.21 (d, 1H, Ar, *J* = 8.5 Hz), 8.75 (d, 1H, Ar, *J* = 2.4 Hz).

**3-(4-Butoxyphenyl)-1-(3-chlorobenzyl)-1-(quinolin-3-yl)urea (3d).** Yield = 44 %; colorless oil. ^1^H NMR (CDCl_3_) *δ* 0.98 (t, 3H, CH_2_*CH_3_*, *J* = 7.4 Hz), 1.48 (sext, 2H, *CH_2_*CH_3_, *J* = 7.6 Hz), 1.75 (quin, 2H, CH_2_*CH_2_*CH_2_, *J* = 7.1 Hz), 3.93 (t, 2H, OCH_2_, *J* = 6.6 Hz), 5.03 (s, 2H, CH_2_N), 6.33 (exch br s, 1H, NH), 6.82 (d, 2H, Ar, *J* = 9.0 Hz), 7.17-7.26 (m, 5H, Ar), 7.35 (s, 1H, Ar), 7.67 (t, 1H, Ar, *J* = 7.8 Hz), 7.79-7.85 (m, 2H, Ar), 8.06 (s, 1H, Ar), 8.19 (d, 1H, Ar, *J* = 8.4 Hz), 8.77 (d, 1H, Ar, *J* = 2.4 Hz).

**2.1.8. General Procedure for 17a,b.**

Et_3_N (1.72 mmol) was added to a suspension of **16** (0.86 mmol), copper acetate (1.29 mmol) and the appropriate butoxyphenylboronic acid (1.72 mmol) in CH_2_Cl_2_ (2 mL). After stirring at room temperature for 15-20 h, the mixture was extracted with a 1:1 solution of 33% aqueous ammonia/saturated aqueous EDTA (3 x 10 mL). The organic layer was washed with 10 mL of water, dried over Na_2_SO_4_ and evaporated. The residue was purified by flash column chromatography using cyclohexane/ethyl acetate 1:3 as eluent.

**Ethyl-1-(4-methoxyphenyl)-7-methyl-4-oxo-1,4-dihydro-1,8-naphthyridine-3-carboxylate (17a).** Yield = 34 %; mp = 146-47 °C (EtOH). ^1^H NMR (CDCl_3_) *δ* 1.42 (t, 3H, CH_2_*CH_3_*, *J* = 7.1 Hz), 2.52 (s, 3H, CH_3_C=N), 3.92 (s, 3H, OCH_3_), 4.41 (q, 2H, OCH_2_, *J* = 7.1 Hz), 7.06 (d, 2H, Ar, *J* = 9.0 Hz), 7.25 (d, 1H, Ar, *J* = 8.1 Hz), 7.35 (d, 2H, Ar, *J* = 8.9 Hz), 8.67 (s, 1H, Ar), 8.69 (d, 1H, Ar, *J* = 8.1 Hz).

**Ethyl-1-(3-methoxyphenyl)-7-methyl-4-oxo-1,4-dihydro-1,8-naphthyridine-3-carboxylate (17b).** Yield = 14 %; mp = 165-66 °C (EtOH). ^1^H NMR (CDCl_3_) *δ* 1.43 (t, 3H, CH_2_*CH_3_*, *J* = 7.1 Hz), 2.54 (s, 3H, CH_3_C=N), 3.89 (s, 3H, OCH_3_), 4.42 (q, 2H, OCH_2_, *J* = 7.1 Hz), 6.99 (t, 1H, Ar, *J* = 2.2 Hz), 7.03 (d, 1H, Ar, *J* = 7.8 Hz), 7.08 (d, 1H, Ar, *J* = 7.7 Hz), 7.27 (d, 1H, Ar, *J* = 8.5 Hz), 7.48 (t, 1H, Ar, *J* = 8.1 Hz), 8.69 (s, 1H, Ar), 8.70 (d, 1H, Ar, *J* = 8.1 Hz).

**2.1.9. General Procedure for 18a,b.**

6N NaOH (3 mL) was added to a suspension of the appropriate intermediate **17a** or **17b** (0.29 mmol) in ethanol (3 mL). After stirring for 0.5-1 h at room temperature, the mixture was concentrated *in vacuo*, diluted with cold water and acidified with 6N HCl. The final product was recovered by filtration and recrystallised from ethanol.

**1-(4-Methoxyphenyl)-7-methyl-4-oxo-1,4-dihydro-1,8-naphthyridine-3-carboxylic acid (18a).** Yield = 99 %; mp = 268-70 °C (EtOH). ^1^H NMR (CDCl_3_) *δ* 2.61 (s, 3H, CH_3_C=N), 3.94 (s, 3H, OCH_3_), 7.08 (d, 2H, Ar, *J* = 9.0 Hz), 7.35 (d, 2H, Ar, *J* = 9.0 Hz), 7.41 (d, 1H, Ar, *J* = 8.2 Hz), 8.73 (d, 1H, Ar, *J* = 8.2 Hz), 8.97 (s, 1H, Ar), 14.60 (exch br s, 1H, COOH).

**1-(3-Methoxyphenyl)-7-methyl-4-oxo-1,4-dihydro-1,8-naphthyridine-3-carboxylic acid (18b).** Yield = 80 %; mp = 217-19 °C (EtOH). ^1^H NMR (CDCl_3_) *δ* 2.61 (s, 3H, CH_3_C=N), 3.90 (s, 3H, OCH_3_), 6.97 (t, 1H, Ar, *J* = 2.2 Hz), 7.01 (d, 1H, Ar, *J* = 7.8 Hz), 7.12 (d, 1H, Ar, *J* = 6.0 Hz), 7.42 (d, 1H, Ar, *J* = 8.2 Hz), 7.50 (t, 1H, Ar, *J* = 8.1 Hz), 8.74 (d, 1H, Ar, *J* = 8.2 Hz), 8.98 (s, 1H, Ar), 14.58 (exch br s, 1H, COOH).

**2.1.10. General Procedure for 4a,b.**

Et_3_N (0.2 mL) was added to a cooled (0 °C) and stirred suspension of **18a** or **18b** (0.19 mmol) in SOCl_2_ (1.5 mL). After 2-3 h at room temperature, the excess of SOCl_2_ was removed *in vacuo*. The intermediates **19a,b** were not isolated and directly dissolved in anhydrous THF (2 mL) at 0 °C. A solution of 4-*n.*butoxyaniline (0.38 mmol) in anhydrous THF (1 mL) was added dropwise and the reaction was carried out at room temperature for 2 h. The mixture was then concentrated under reduced pressure. After dilution with ice-cold water (10 mL), the precipitate was filtered and purified by flash column chromatography using cyclohexane/ethyl acetate 2:1 (for **4a**) or cyclohexane/ethyl acetate 1:1 (for **4b**) as eluents.

***N*-(4-Butoxyphenyl)-1-(4-methoxyphenyl)-7-methyl-4-oxo-1,4-dihydro-1,8-naphthyridine-3-carboxamide (4a).** Yield = 35 %; mp = 154-55 °C (EtOH). ^1^H NMR (CDCl_3_) *δ* 1.00 (t, 3H, CH_2_*CH_3_*, *J* = 7.4 Hz), 1.52 (sext, 2H, *CH_2_*CH_3_, *J* = 7.6 Hz), 1.79 (quin, 2H, CH_2_*CH_2_*CH_2_, *J* = 7.9 Hz), 2.58 (s, 3H, CH_3_C=N), 3.94 (s, 3H, OCH_3_), 3.99 (t, 2H, OCH_2_, *J* = 6.5 Hz), 6.92 (d, 2H, Ar, *J* = 9.0 Hz), 7.07 (d, 2H, Ar, *J* = 8.9 Hz), 7.35 (d, 1H, Ar, *J* = 8.2 Hz), 7.38 (d, 2H, Ar, *J* = 8.9 Hz), 7.70 (d, 2H, Ar, *J* = 9.0 Hz), 8.74 (d, 1H, Ar, *J* = 8.2 Hz), 9.08 (s, 1H, Ar), 11.97 (exch br s, 1H, NH).

***N*-(4-Butoxyphenyl)-1-(3-methoxyphenyl)-7-methyl-4-oxo-1,4-dihydro-1,8-naphthyridine-3-carboxamide (4b).** Yield = 44 %; mp = 203-05 °C (EtOH). ^1^H NMR (CDCl_3_) *δ* 1.00 (t, 3H, CH_2_*CH_3_*, *J* = 7.4 Hz), 1.52 (sext, 2H, *CH_2_*CH_3_, *J* = 7.6 Hz), 1.79 (quin, 2H, CH_2_*CH_2_*CH_2_, *J* = 7.1 Hz), 2.59 (s, 3H, CH_3_C=N), 3.89 (s, 3H, OCH_3_), 3.99 (t, 2H, OCH_2_, *J* = 6.5 Hz), 6.92 (d, 2H, Ar, *J* = 9.0 Hz), 7.01 (s, 1H, Ar), 7.05 (d, 1H, Ar, *J* = 7.8 Hz), 7.10 (d, 1H, Ar, *J* = 7.7 Hz), 7.35 (d, 1H, Ar, *J* = 8.2 Hz), 7.48 (t, 1H, Ar, *J* = 8.1 Hz), 7.70 (d, 2H, Ar, *J* = 9.0 Hz), 8.74 (d, 1H, Ar, *J* = 8.2 Hz), 9.09 (s, 1H, Ar), 11.95 (exch br s, 1H, NH).

**2.1.11. General Procedure for 4c,d.**

Et_3_N (0.2 mL) was added to a cooled (0 °C) and stirred suspension of **18a** or **18b** (0.29 mmol) in SOCl_2_ (1.5 mL). After 2-3 h at 60 °C, the mixture was allowed to cool down and the excess of SOCl_2_ was removed *in vacuo*. The intermediates **20a,b** were not isolated and directly dissolved in anhydrous THF (1 mL) at 0 °C. A solution of 4-*n.*Butoxyaniline (0.58 mmol) in anhydrous THF (1 mL) was added dropwise and the reaction was carried out at room temperature for 2 h. The mixture was then concentrated in vacuo and diluted with ice-cold water (10 mL). The precipitate was filtered and purified by flash column chromatography using CH_2_Cl_2_ (for **4c**) or CH_2_Cl_2_/MeOH 99:1 (for **4d**) as eluents.

***N*-(4-Butoxyphenyl)-1-(4-methoxyphenyl)-4-oxo-7-trichloromethyl-1,4-dihydro-1,8-naphthyridine-3-carboxamide (4c).** Yield = 49 %; mp = 289-91 °C (EtOH). ^1^H NMR (CDCl_3_) *δ* 1.01 (t, 3H, CH_2_*CH_3_*, *J* = 7.4 Hz), 1.52 (sext, 2H, *CH_2_*CH_3_, *J* = 7.6 Hz), 1.81 (quin, 2H, CH_2_*CH_2_*CH_2_, *J* = 6.6 Hz), 3.94 (s, 3H, OCH_3_), 4.00 (t, 2H, OCH_2_, *J* = 6.5 Hz), 6.93 (d, 2H, Ar, *J* = 9.0 Hz), 7.07 (d, 2H, Ar, *J* = 8.9 Hz), 7.44 (d, 2H, Ar, *J* = 8.9 Hz), 7.70 (d, 2H, Ar, *J* = 9.00 Hz), 8.15 (d, 1H, Ar, *J* = 8.4 Hz), 9.02 (d, 1H, Ar, *J* = 8.4 Hz), 9.20 (s, 1H, Ar), 11.75 (exch br s, 1H, NH). MS (ESI) calcd. for C_27_H_24_Cl_3_N_3_O_4_, 560.86; found: *m/z* 560.36 [M]^+^, 562.36 [M + H]^+^.

***N*-(4-Butoxyphenyl)-1-(3-methoxyphenyl)-4-oxo-7-trichloromethyl-1,4-dihydro-1,8-naphthyridine-3-carboxamide (4d).** Yield = 54 %; mp = 148-50 °C (EtOH). ^1^H NMR (CDCl_3_) *δ* 1.01 (t, 3H, CH_2_*CH_3_*, *J* = 7.4 Hz), 1.52 (sext, 2H, *CH_2_*CH_3_, *J* = 7.6 Hz), 1.80 (quin, 2H, CH_2_*CH_2_*CH_2_, *J* = 6.8 Hz), 3.88 (s, 3H, OCH_3_), 3.99 (t, 2H, OCH_2_, *J* = 6.5 Hz), 6.93 (d, 2H, Ar, *J* = 8.9 Hz), 7.08 (s, 1H, Ar), 7.10 (d, 2H, Ar, *J* = 8.1 Hz), 7.49 (t, 1H, Ar, *J* = 8.1 Hz), 7.70 (d, 2H, Ar, *J* = 8.9 Hz), 8.15 (d, 1H, Ar, *J* = 8.4 Hz), 9.02 (d, 1H, Ar, *J* = 8.4 Hz), 9.23 (s, 1H, Ar), 11.73 (exch br s, 1H, NH). MS (ESI) calcd. for C_27_H_24_Cl_3_N_3_O_4_, 560.86: found: *m/z* 560.36 [M]^+^, 562.36 [M + H]^+^, 584.18 [M + Na]^+^.

**2.1.12. General Procedure for 21a-c.**

A mixture of **16** (0.65 mmol), K_2_CO_3_ (1.30 mmol) and the appropriate benzyl halide (0.71 mmol) was stirred in anhydrous acetone (3 mL) under reflux for 5-7 h. The mixture was then concentrated *in vacuo*, diluted with cold water and extracted with CH_2_Cl_2_ (3 x 15 mL). The organic layer was dried over Na_2_SO_4_ and evaporated. Compounds **21a-c** were purified by column chromatography using cyclohexane/ethyl acetate 1:6 (for **21a,b**) or cyclohexane/ethyl acetate 1:9 (for **21c**) as eluents.

**Ethyl-1-(4-methoxybenzyl)-7-methyl-4-oxo-1,4-dihydro-1,8-naphthyridine-3-carboxylate (21a).** Yield = 35 %; mp = 107-09 °C (EtOH). ^1^H NMR (CDCl_3_) *δ* 1.41 (t, 3H, CH_2_*CH_3_*, *J* = 7.1 Hz), 2.69 (s, 3H, CH_3_C=N), 3.80 (s, 3H, OCH_3_), 4.40 (q, 2H, OCH_2_, *J* = 7.1 Hz), 5.59 (s, 2H, NCH_2_), 6.88 (d, 2H, Ar, *J* = 8.6 Hz), 7.26 (d, 1H, Ar, *J* = 8.1 Hz), 7.32 (d, 2H, Ar, *J* = 8.1 Hz), 8.66 (d, 1H, Ar, *J* = 8.1 Hz), 8.71 (s, 1H, Ar).

**Ethyl-1-(3-methoxybenzyl)-7-methyl-4-oxo-1,4-dihydro-1,8-naphthyridine-3-carboxylate (21b).** Yield = 18 %; colorless oil. ^1^H NMR (CDCl_3_) *δ* 1.43 (t, 3H, CH_2_*CH_3_*, *J* = 7.0 Hz), 2.69 (s, 3H, CH_3_C=N), 3.80 (s, 3H, OCH_3_), 4.43 (q, 2H, OCH_2_, *J* = 7.1 Hz), 5.69 (s, 2H, NCH_2_), 6.87 (d, 1H, Ar, *J* = 8.5 Hz), 6.92 (s, 1H, Ar), 6.93 (d, 1H, Ar, *J* = 8.2 Hz), 7.26-7.31 (m, 2H, Ar), 8.68 (d, 1H, Ar, *J* = 8.0 Hz), 8.71 (s, 1H, Ar).

**Ethyl-1-(1,3-benzodioxol-5-ylmethyl)-7-methyl-4-oxo-1,4-dihydro-1,8-naphthyridine-3-carboxylate (21c).** Yield = 70 %; mp = 152-54 °C (EtOH). ^1^H NMR (CDCl_3_) *δ* 1.42 (t, 3H, CH_2_*CH_3_*, *J* = 7.1 Hz), 2.69 (s, 3H, CH_3_C=N), 4.40 (q, 2H, OCH_2_, *J* = 7.1 Hz), 5.54 (s, 2H, NCH_2_), 5.96 (s, 2H, OCH_2_O), 6.78 (d, 1H, Ar, *J* = 7.7 Hz), 6.87 (d, 2H, Ar, *J* = 8.2 Hz), 7.28 (d, 1H, Ar, *J* = 3.0 Hz), 8.66 (d, 2H, Ar, *J* = 7.3 Hz).

**2.1.13. General Procedure for 22a-c.**

6N NaOH (6 mL) was added to a suspension of the appropriate derivative **21a-c** (0.65 mmol) in ethanol (5 mL) and the reaction was stirred for 2 h at room temperature. The mixture was concentrated *in vacuo*, diluted with cold water and acidified with 6N HCl. The pure compounds **22a-c** were obtained by filtration and recrystallisation from ethanol.

**1-(4-Methoxybenzyl)-7-methyl-4-oxo-1,4-dihydro-1,8-naphthyridine-3-carboxylic acid (22a).** Yield = 67 %; mp = 215-17 °C (EtOH). ^1^H NMR (CDCl_3_) *δ* 2.79 (s, 3H, CH_3_C=N), 3.81 (s, 3H, OCH_3_), 5.69 (s, 2H, NCH_2_), 6.89 (d, 2H, Ar, *J* = 8.5 Hz), 7.36 (d, 2H, Ar, *J* = 8.5 Hz), 7.42 (d, 1H, Ar, *J* = 8.2 Hz), 8.70 (d, 1H, Ar, *J* = 8.2 Hz), 8.97 (s, 1H, Ar), 14.63 (exch br s, 1H, OH).

**1-(3-Methoxybenzyl)-7-methyl-4-oxo-1,4-dihydro-1,8-naphthyridine-3-carboxylic acid (22b).** Yield = 77 %; mp = 244-46 °C (EtOH). ^1^H NMR (CDCl_3_) *δ* 2.77 (s, 3H, CH_3_C=N), 3.80 (s, 3H, OCH_3_), 5.73 (s, 2H, NCH_2_), 6.87-6.91 (m, 2H, Ar), 6.93 (d, 1H, Ar, *J* = 7.7 Hz), 7.29 (t, 2H, Ar, *J* = 8.1 Hz), 7.42 (d, 1H, Ar, *J* = 8.2 Hz), 8.71 (d, 1H, Ar, *J* = 8.2 Hz), 8.97 (s, 1H, Ar), 14.60 (exch br s, 1H, OH).

**1-(1,3-Benzodioxol-5-ylmethyl)-7-methyl-4-oxo-1,4-dihydro-1,8-naphthyridine-3-carboxylic acid (22c).** Yield = 68 %; mp = 237-39 °C (EtOH). ^1^H NMR (CDCl_3_) *δ* 2.79 (s, 3H, CH_3_C=N), 5.66 (s, 2H, NCH_3_), 5.98 (s, 2H, OCH_2_O), 6.80 (d, 1H, Ar, *J* = 7.9 Hz), 6.88 (s, 1H, Ar), 6.90 (d, 1H, Ar, *J* = 7.9 Hz), 7.43 (d, 1H, Ar, *J* = 8.2 Hz), 8.70 (d, 1H, Ar, *J* = 8.2 Hz), 8.95 (s, 1H, Ar).

**2.1.14. General Procedure for 4e-g.**

Et_3_N (5 drops), diethyl-cyanophosphonate (0.68 mmol) and 4-*n.*butoxyaniline (0.17 mmol) were added to a cooled (0 °C) and stirred solution of the suitable intermediate **22a-c** (0.17 mmol) in anhydrous DMF (1 mL). After 0.5 h at 0 °C, the reaction was carried out at room temperature for 15 h. The mixture was then diluted with ice-cold water (10 mL) and kept under stirring for 0.5 h at 0 °C. The precipitate was obtained by filtration and purified by flash column chromatography using cyclohexane/ethyl acetate 2:1 as eluent.

***N*-(4-Butoxyphenyl)-1-(4-methoxybenzyl)-7-methyl-4-oxo-1,4-dihydro-1,8-naphthyridine-3-carboxamide (4e).** Yield = 75 %; mp = 190-91 °C (EtOH). ^1^H NMR (CDCl_3_) *δ* 1.00 (t, 3H, CH_2_*CH_3_*, *J* = 7.4 Hz), 1.51 (sext, 2H, *CH_2_*CH_3_, *J* = 7.6 Hz), 1.79 (quin, 2H, CH_2_*CH_2_*CH_2_, *J* = 7.0 Hz), 2.75 (s, 3H, CH_3_C=N), 3.80 (s, 3H, OCH_3_), 3.98 (t, 2H, OCH_2_, *J* = 6.5 Hz), 5.68 (s, 2H, NCH_2_), 6.89 (qd, 4H, Ar, *J* = 3.5 Hz, *J* = 9.0 Hz), 7.34-7.37 (m, 3H, Ar), 7.68 (d, 2H, Ar, *J* = 7.9 Hz), 8.69 (d, 1H, Ar, *J* = 8.1 Hz), 9.08 (s, 1H, Ar), 11.97 (exch br s, 1H, NH).

***N*-(4-Butoxyphenyl)-1-(3-methoxybenzyl)-7-methyl-4-oxo-1,4-dihydro-1,8-naphthyridine-3-carboxamide (4f).** Yield = 71 %; mp = 145-47 °C (EtOH). ^1^H NMR (CDCl_3_) *δ* 1.00 (t, 3H, CH_2_*CH_3_*, *J* = 7.4 Hz), 1.51 (sext, 2H, *CH_2_*CH_3_, *J* = 7.6 Hz), 1.79 (quin, 2H, CH_2_*CH_2_*CH_2_, *J* = 6.9 Hz), 2.74 (s, 3H, CH_3_C=N), 3.80 (s, 3H, OCH_3_), 3.98 (t, 2H, OCH_2_, *J* = 6.5 Hz), 5.72 (s, 2H, NCH_2_), 6.86 (d, 1H, Ar, *J* = 8.5 Hz), 6.91 (d, 2H, Ar, *J* = 8.9 Hz), 6.95-7.02 (m, 2H, Ar), 7.27 (t, 1H, Ar, *J* = 7.9 Hz), 7.37 (d, 1H, Ar, *J* = 7.0 Hz), 7.69 (d, 2H, Ar, *J* = 8.2 Hz), 8.71 (d, 1H, Ar, *J* = 8.2 Hz), 9.08 (s, 1H, Ar), 11.97 (exch br s, 1H, NH).

**1-(1,3-Benzodioxol-5-ylmethyl)-*N*-(4-butoxyphenyl)-7-methyl-4-oxo-1,4-dihydro-1,8-naphthyridine-3-carboxamide (4g).** Yield = 62 %; mp = 181-83 °C (EtOH). ^1^H NMR (CDCl_3_) *δ* 1.00 (t, 3H, CH_2_*CH_3_*, *J* = 7.3 Hz), 1.51 (sext, 2H, *CH_2_*CH_3_, *J* = 7.8 Hz), 1.79 (quin, 2H, CH_2_*CH_2_*CH_2_, *J* = 6.8 Hz), 2.77 (s, 3H, CH_3_C=N), 3.98 (t, 2H, OCH_2_, *J* = 6.4 Hz), 5.65 (s, 2H, NCH_2_), 5.96 (s, 2H, OCH_2_O), 6.79 (d, 1H, Ar, *J* = 7.8 Hz), 6.91 (d, 4H, Ar, *J* = 8.5 Hz), 7.38 (d, 1H, Ar, *J* = 6.7 Hz), 7.69 (d, 2H, Ar, *J* = 5.6 Hz), 8.71 (d, 1H, Ar, *J* = 8.0 Hz), 9.06 (s, 1H, Ar), 11.97 (exch br s, 1H, NH).

**2.1.15. *N*-(4-Bromophenyl)-1-ethyl-7-methyl-4-oxo-1,4-dihydro-1,8-naphthyridine-3-carboxamide (4h).**

Et_3_N (1.50 mmol) was added to a cooled (-5 °C) and stirred solution of nalidixic acid **23** (0.43 mmol) in anhydrous tetrahydrofuran (3 mL). After 30 min, the mixture was allowed to warm up to 0 °C and ethyl chloroformate (0.47 mmol) was added. After 1 h 4-bromoaniline was added and the reaction was carried out at room temperature for 12 h. The mixture was then concentrated *in vacuo*, diluted with cold water (20 mL) and extracted with CH_2_Cl_2_ (3 x 15 mL). The solvent was evaporated to afford final compound **4h**, which was purified by two consecutive flash chromatography using cyclohexane/ethyl acetate 2:1 and CH_2_Cl_2_ as eluents. Yield = 18 %; mp = 290-92 °C (EtOH). ^1^H NMR (CDCl_3_) *δ* 1.56 (t, 3H, CH_2_*CH_3_*, *J* = 7.2 Hz), 2.74 (s, 3H, CH_3_C=N), 4.62 (q, 2H, NCH_2_, *J* = 7.2 Hz), 7.97 (d, 1H, Ar, *J* = 8.2 Hz), 7.48 (d, 2H, Ar, *J* = 8.7 Hz ), 7.70 (d, 2H, Ar, *J* = 8.8 Hz), 8.70 (d, 1H, Ar, *J* = 8.1 Hz), 9.00 (s, 1H, Ar), 12.24 (exch br s, 1H, NH).

**2.1.16. *N*-(4-Butoxyphenyl)-1-ethyl-4-oxo-7-trichloromethyl-1,4-dihydro-1,8-naphthyridine-3-carboxamide (4j).**

Et_3_N (0.2 mL) was added to a cooled (0 °C) and stirred suspension of nalidixic acid (0.43 mmol) in SOCl_2_ (1.5 mL). After 2 h at 60 °C, the mixture was allowed to cool down and the excess of SOCl_2_ was removed *in vacuo*. The residue was dissolved in anhydrous THF (3 mL) and cooled again to 0 °C. A solution of 4-*n.*butoxyaniline (0.86 mmol) in anhydrous THF (2 mL) was then added dropwise and the reaction was carried out at room temperature for 2 h. The mixture was concentrated under reduced pressure and diluted with ice-cold water (10 mL). The precipitate was recovered by filtration and purified by flash column chromatography using CH_2_Cl_2_/MeOH/CH_3_COOH 99:1:0.1 as eluent. Yield = 15 %; mp = 162-63 °C (EtOH). ^1^H NMR (CDCl_3_) *δ* 1.01 (t, 3H, CH_2_CH_2_*CH_3_*, *J* = 7.4 Hz), 1.52 (sext, 2H, CH_2_*CH_2_*CH_3_, *J* = 7.6 Hz), 1.62 (t, 3H, NCH_2_*CH_3_*, *J* = 7.2 Hz), 1.80 (quin, 2H, CH_2_*CH_2_*CH_2_, *J* = 7.1 Hz), 4.00 (t, 2H, OCH_2_, *J* = 6.6 Hz), 4.64 (q, 2H, NCH_2_, *J* = 7.2 Hz), 6.93 (d, 2H, Ar, *J* = 9.0 Hz), 7.69 (d, 2H, Ar, *J* = 9.0 Hz), 8.15 (d, 1H, Ar, *J* = 8.4 Hz), 8.99 (d, 1H, Ar, *J* = 8.4 Hz ), 9.12 (s, 1H, Ar), 11.76 (exch br s, 1H, NH).

**2.1.17. *N*-(4-Bromophenyl)-2-(1-oxophthalazin-2(1*H*)-yl)acetamide (5).**

A solution of intermediate **24** (1.54 mmol) in anhydrous acetonitrile (1 ml) was added dropwise to a stirred solution of the commercially available 1-(2*H*)-phthalazinone (1.03 mmol) in anhydrous acetonitrile (3 mL). K_2_CO_3_ (2.06 mmol) was added and the reaction was carried out for 3 h at reflux. Removal of the solvent gave a residue which was poured into ice-cold water. After 1 h stirring in ice-bath the product was recovered by filtration and recrystallised from ethanol. Yield = 45 %; mp = 245-46 °C (EtOH). ^1^H NMR (CDCl_3_) *δ* 4.97 (s, 2H, NCH_2_CO), 7.53 (qd, 4H, Ar, *J* = 13.9 Hz, *J* = 8.3 Hz), 7.91 (m, 1H, Ar), 7.99 (d, 2H, Ar, *J* = 5.8 Hz), 8.26 (d, 1H, Ar, *J* = 8.0 Hz), 8.48 (s, 1H, Ar), 10.46 (exch br s, 1H, NH).

**2.1.18. General Procedure for 26a-c.**

Commercially available 2,3-diidrophthalazin-1,4-dione **25** (4.93 mmol), K_2_CO_3_ (9.80 mmol) and the suitable benzyl halide (5.43 mmol) were stirred in anhydrous DMF (5 mL) for 2-4 h at 80 °C. The mixture was then diluted with ice-cold water and extracted with CH_2_Cl_2_ (3 x 15 mL). The organic layer was dried over Na_2_SO_4_ and evaporated *in vacuo*. Intermediates **26a-c** were then purified by flash column chromatography using cyclohexane/ethyl acetate 1:1 as eluent.

**2-(4-Methoxybenzyl)-2,3-dihydrophthalazine-1,4-dione (26a).** Yield = 23 %; mp = 150-52 °C (EtOH). ^1^H NMR (CDCl_3_) *δ* 3.86 (s, 3H, OCH_3_), 5.31 (s, 2H, NCH_2_), 6.96 (d, 2H, Ar, *J* = 8.6 Hz), 7.44 (d, 2H, Ar, *J* = 8.5 Hz), 7.83 (quin, 2H, Ar, *J* = 3.7 Hz), 8.07 (dd, 1H, Ar, *J* = 3.1 Hz, *J* = 3.5 Hz), 8.41 (dd, 1H, Ar, *J* = 2.4 Hz, *J* = 2.8 Hz), 9.98 (exch br s, 1H, NH).

**2-(3-Methoxybenzyl)-2,3-dihydrophthalazine-1,4-dione (26b).** Yield = 18 %; mp = 184-86 °C (EtOH). ^1^H NMR (CDCl_3_) *δ* 3.87 (s, 3H, OCH_3_), 5.36 (s, 2H, NCH_2_), 6.93 (dd, 1H, Ar, *J* = 5.8 Hz, *J* = 2.4 Hz), 7.06 (s, 1H, Ar), 7.09 (d, 1H, Ar, *J* = 7.6 Hz), 7.36 (t, 1H, Ar, *J* = 7.9 Hz), 7.82-7.89 (m, 2H, Ar), 8.09 (dd, 1H, Ar, *J* = 6.9 Hz, *J* = 1.9 Hz), 8.44 (dd, 1H, Ar, *J* = 4.0 Hz, *J* = 2.1 Hz), 9.94 (exch br s, 1H, NH).

**2.1.19. General Procedure for 6a-c.**

The appropriate intermediate **26a-c** (1.10 mmol) was dissolved in 3 mL of anhydrous CH_2_Cl_2_ and 4-butoxyphenyl isocyanate (2.20 mmol) was added under stirring at 0 °C. The reaction was carried out at 0 °C for 2 h and at room temperature for 12 h. The solid residue was filtered off and the solution was evaporated *in vacuo* to afford compounds **6a-c**, which were purified by flash column chromatography using CH_2_Cl_2_ as eluent.

***N*-(4-Butoxyphenyl)-3-(4-methoxybenzyl)-1,4-dioxo-3,4-dihydro-phthalazine-2(1*H*)-carboxamide (6a).** Yield = 53 %; mp = 117-19 °C (EtOH). ^1^H NMR (CDCl_3_) *δ* 1.01 (t, 3H, CH_2_*CH_3_*, *J* = 7.4 Hz), 1.52 (sext, 2H, *CH_2_*CH_3_, *J* = 7.5 Hz), 1.80 (quin, 2H, CH_2_*CH_2_*CH_2_, *J* = 6.8 Hz), 3.84 (s, 3H, OCH_3_), 3.99 (t, 2H, OCH_2_, *J* = 6.5 Hz), 5.51 (s, 2H, NCH_2_), 6.95 (t, 4H, Ar, *J* = 8.7 Hz), 7.56 (d, 2H, Ar, *J* = 8.5 Hz), 7.62 (d, 2H, Ar, *J* = 8.9 Hz), 7.86 (quin, 2H, Ar, *J* = 7.8 Hz), 8.06 (d, 1H, Ar, *J* = 7.8 Hz), 8.47 (d, 1H, Ar, *J* = 7.6 Hz), 11.86 (exch br s, 1H, NH).

***N*-(4-Butoxyphenyl)-3-(3-methoxybenzyl)-1,4-dioxo-3,4-dihydro-phthalazine-2(1*H*)-carboxamide (6b).** Yield = 75 %; mp = 104-06 °C (EtOH). ^1^H NMR (CDCl_3_) *δ* 1.01 (t, 3H, CH_2_*CH_3_*, *J* = 7.4 Hz), 1.52 (sext, 2H, *CH_2_*CH_3_, *J* = 7.6 Hz), 1.80 (quin, 2H, CH_2_*CH_2_*CH_2_, *J* = 7.0 Hz), 3.87 (s, 3H, OCH_3_), 3.99 (t, 2H, OCH_2_, *J* = 6.5 Hz), 5.56 (s, 2H, NCH_2_), 6.93 (dd, 3H, Ar, *J* = 4.6 Hz, *J* = 3.2 Hz), 7.18 (d, 1H, Ar, *J* = 7.0 Hz), 7.19 (s, 1H, Ar), 7.35 (t, 1H, Ar, *J* = 7.7 Hz), 7.61 (d, 2H, Ar, *J* = 9.0 Hz), 7.84-7.92 (m, 2H, Ar), 8.10 (d, 1H, Ar, *J* = 7.1 Hz), 8.49 (d, 1H, Ar, *J* = 7.2 Hz), 11.84 (exch br s, 1H, NH).

**3-Benzyl-*N*-(4-butoxyphenyl)-1,4-dioxo-3,4-dihydrophthalazine-2(1*H*)-carboxamide (6c).** Yield = 86 %; mp = 117-19 °C (EtOH). ^1^H NMR (CDCl_3_) *δ* 1.01 (t, 3H, CH_2_*CH_3_*, *J* = 7.4 Hz), 1.52 (sext, 2H, *CH_2_*CH_3_, *J* = 7.6 Hz), 1.80 (quin, 2H, CH_2_*CH_2_*CH_2_, *J* = 6.9 Hz), 3.99 (t, 2H, OCH_2_, *J* = 6.5 Hz), 5.59 (s, 2H, NCH_2_), 6.94 (d, 2H, Ar, *J* = 8.9 Hz), 7.37-7.46 (m, 3H, Ar), 7.62 (dd, 4H, Ar, *J* = 1.3 Hz, *J* = 5.3 Hz), 7.84-7.92 (m, 2H, Ar), 8.09 (d, 1H, Ar, *J* = 7.8 Hz), 8.49 (d, 1H, Ar, *J* = 7.5 Hz), 11.85 (exch br s, 1H, NH).

**2.1.20. *N*-(4-Bromophenyl)-2-[3-(3-methoxybenzyl)-1,4-dioxo-3,4-dihydro-phthalazin-2(1*H*)-yl]acetamide (6d).**

A suspension of **26b** (0.18 mmol), K_2_CO_3_ (0.36 mmol) and **24** (0.27 mmol) was stirred in anhydrous acetonitrile (1 mL) at reflux for 3 h. The solvent was then evaporated *in vacuo* and the mixture was poured into ice-cold water. After 1 h stirring in ice-bath, the precipitate was removed by filtration and purified through two consecutive crystallisation from ethanol. Yield = 45 %; mp = 178-80 °C (EtOH). ^1^H NMR (CDCl_3_) *δ* 3.85 (s, 3H, OCH_3_), 4.95 (s, 2H, N*CH_2_*-C_6_H_3_), 5.39 (s, 2H, NCH_2_CO), 6.91 (d, 1H, Ar, *J* = 8.5 Hz), 7.06 (s, 1H, Ar), 7.09 (d, 1H, Ar, *J* = 6.7 Hz), 7.34 (t, 1H, Ar, *J* = 7.6 Hz), 7.43 (s, 4H, Ar), 7.78 (t, 2H, Ar, *J* = 4.8 Hz), 8.09-8.12 (m, 1H, Ar), 8.45-8.48 (m, 1H, Ar), 8.80 (exch br s, 1H, NH).

**3. Experimental Details - Molecular modelling**

The 2D chemical structures were constructed by Marvin Sketch and were all subjected to molecular mechanics energy minimisation using the MMFF94 force field present in the same software. Once obtained the 3D structures for all compounds, the geometry was also optimised at semi-empirical level using the PM3 semi-empirical Hamiltonian as implemented in MOPAC 2016 package. The database of protein targets was built through a selection of crystallised structures in the Protein Data Bank database (www.rcsb.org). Water molecules were removed and polar hydrogens were added with Autodock-Tools 1.4.5. Molecular docking calculations were performed using Autodock-Vina software. In the configuration file, we fixed the exhaustiveness value at 25 and the coordinate values for the targets, by focusing the grids on the binding sites of the co-crystallised ligand. Autodock-Vina results were analysed with Autodock Tools 1.4.5.

**Table 1S**. Selected protein targets for the iVS.

| PDB code^a^ | Protein name |
| --- | --- |
| 2xab | HSP90 |
| 3d0e | AKT kinase |
| 3l3l | poly(ADP-ribose) polymerase (PARP) |
| 3oyw | galectin-1 |
| 4ase | VEGFR2 |
| 4qmz | Mammalian Sterile20-like Kinase 3 (MST3) |
| 1pkd | Phospho-CDK2/Cyclin A |
| 1xkk | EGFR kinase |
| 2fb8 | B-Raf kinase |
| 2nnq | Fatty acid binding protein 4 |
| 2rku | PLK1 |
| 3hb4 | 17beta-hydroxysteroid dehydrogenase type1 |
| 3l08 | Pi3K gamma |
| 3lbz | BCL6 |
| 3pe1 | protein kinase CK2 |
| 3zxz | c-Met |
| 4fod | human anaplastic lymphoma kinase |
| 4ks8 | PAK6 kinase |
| 4mdn | MDM2 |
| 4o9v | matriptase |
| 4p5e | DNPH1 |
| 4pp3 | human Retinoid X Receptor alpha |
| 4q07 | carbonic anhydrase IX |
| 4u5j | c-Src |
| 4ual | MRCK beta |
| 4v05 | FGFR1 |
| 5h2u | PTK6 |
| 4uxl | Human ROS1 Kinase |
| 4yc8 | c-Abl Kinase |
| 5hi2 | BRAF |
| 5i9y | Ephrin A2 Receptor Protein Kinase |
| 5jrq | BRAFV600E |

^a^ Downloaded from <https://www.rcsb.org/>

**Table 2S**. Δ*G* results of the iVS calculations.

**Table 3S**. Results of calculated V values (using eq. 2).

**Figure 1S**. V values of co-crystallised ligand and screened compounds for **2xab** protein.

**Figure 2S**. V values of co-crystallised ligand and screened compounds for **3d0e** protein.

**Figure 3S**. V values of co-crystallised ligand and screened compounds for **3l3l** protein.

**Figure 4S**. V values of co-crystallised ligand and screened compounds for **3oyw** protein.

**Figure 5S**. V values of co-crystallised ligand and screened compounds for **4ase** protein.

**Figure 6S**. V values of co-crystallised ligand and screened compounds for **4qmz** protein.

**Figure 7S**. V values of co-crystallised ligand and screened compounds for **1pkd** protein.

**Figure 8S**. V values of co-crystallised ligand and screened compounds for **1xkk** protein.

**Figure 9S**. V values of co-crystallised ligand and screened compounds for **2fb8** protein.

**Figure 10S**. V values of co-crystallised ligand and screened compounds for **2nnq** protein.

**Figure 11S**. V values of co-crystallised ligand and screened compounds for **2rku** protein.

**Figure 12S**. V values of co-crystallised ligand and screened compounds for **3hb4** protein.

**Figure 13S**. V values of co-crystallised ligand and screened compounds for **3l08** protein.

**Figure 14S**. V values of co-crystallised ligand and screened compounds for **3lbz** protein.

**Figure 15S**. V values of co-crystallised ligand and screened compounds for **3pe1** protein.

**Figure 16S**. V values of co-crystallised ligand and screened compounds for **3zxz** protein.

**Figure 17S**. V values of co-crystallised ligand and screened compounds for **4fod** protein.

**Figure 18S**. V values of co-crystallised ligand and screened compounds for **4ks8** protein.

**Figure 19S**. V values of co-crystallised ligand and screened compounds for **4mdn** protein.

**Figure 20S**. V values of co-crystallised ligand and screened compounds for **4o9v** protein.

**Figure 21S**. V values of co-crystallised ligand and screened compounds for **4p5e** protein.

**Figure 22S**. V values of co-crystallised ligand and screened compounds for **4pp3** protein.

**Figure 23S**. V values of co-crystallised ligand and screened compounds for **4q07** protein.

**Figure 24S**. V values of co-crystallised ligand and screened compounds for **4u5j** protein.

**Figure 25S**. V values of co-crystallised ligand and screened compounds for **4ual** protein.

**Figure 26S**. V values of co-crystallised ligand and screened compounds for **4v05** protein.

**Figure 27S**. V values of co-crystallised ligand and screened compounds for **5h2u** protein.

**Figure 28S**. V values of co-crystallised ligand and screened compounds for **4uxl** protein.

**Figure 29S**. V values of co-crystallised ligand and screened compounds for **4yc8** protein.

**Figure 30S**. V values of co-crystallised ligand and screened compounds for **5hi2** protein.

**Figure 31S**. V values of co-crystallised ligand and screened compounds for **5i9y** protein.

**Figure 32S**. V values of co-crystallised ligand and screened compounds for **5jrq** protein.

**Table 4S**. Results of the toxicity risk assessment and the fragment based druglikeness.

|  | Druglikeness | Mutagenic | Tumorigenic | Reproductive Effects | Irritant | DrugScore |
| --- | --- | --- | --- | --- | --- | --- |
| **1a** | -0.47737 | none | none | high | high | 0.125148 |
| **1b** | -0.47737 | none | none | high | high | 0.125148 |
| **1c** | 0.093731 | none | none | high | high | 0.134076 |
| **1d** | 0.093731 | none | none | high | high | 0.134076 |
| **1e** | 0.092176 | none | none | high | high | 0.10526 |
| **1f** | 0.01431 | none | none | high | high | 0.111798 |
| **2a** | -2.1291 | none | none | none | high | 0.105097 |
| **2b** | -2.1291 | none | none | none | high | 0.105097 |
| **2c** | -1.1717 | none | none | none | high | 0.163252 |
| **2d** | -1.1717 | none | none | none | high | 0.163252 |
| **2e** | -1.1833 | none | none | none | high | 0.128609 |
| **2f** | -1.2644 | none | none | none | high | 0.135928 |
| **2g** | 1.0431 | none | high | none | none | 0.171588 |
| **2h** | 1.6423 | none | high | none | none | 0.128485 |
| **3a** | -2.4198 | none | none | none | high | 0.099535 |
| **3b** | -2.4198 | none | none | none | high | 0.099535 |
| **3c** | -2.5278 | none | none | none | high | 0.087365 |
| **3d** | -2.443 | none | none | none | high | 0.083515 |
| **4a** | -1.565 | none | high | none | high | 0.076344 |
| **4b** | -1.565 | none | high | none | high | 0.076344 |
| **4c** | -1.6252 | high | high | high | high | 0.015771 |
| **4d** | -1.6252 | high | high | high | high | 0.015771 |
| **4e** | -0.64766 | none | high | none | high | 0.099147 |
| **4f** | -0.64766 | none | high | none | high | 0.099147 |
| **4g** | -0.73678 | none | high | none | high | 0.084656 |
| **4h** | 2.6254 | none | high | none | none | 0.368462 |
| **4j** | -0.98071 | high | high | high | high | 0.026785 |
| **5** | 3.5414 | none | high | none | none | 0.442036 |
| **6a** | -2.0216 | none | none | none | high | 0.16807 |
| **6b** | -2.0216 | none | none | none | high | 0.16807 |
| **6c** | -2.1186 | none | none | none | high | 0.175 |
| **6d** | 1.7672 | none | high | none | none | 0.323085 |

**References**

1. El-Gendy, A. A.; Osman, A. N.; Khalifa, M. Synthesis of 3-Indoleacetyl Derivatives of Certain Amino and Phenolic Compounds Likely to Possess Antiinflammatory Activity. Pharmazie **1982**, 37, 481-482.
2. Lappin, G.R. Cyclization of 2-aminopyridine derivatives. I. Substituted ethyl 2-pyridylaminomethylenemalonates. J. Am. Chem. Soc. **1948**, 70, 3348–3350.
3. Hermecz, I.; Mészáros, Z.; Vasvári-Debreczy, L.; Horváth, A.; Horváth, G.; Pongor-Csákvári M. Nitrogen bridgehead compounds. Part 4. 1 → 3 N→C-acyl migration. Part 2. J. Chem. Soc., Perkin Trans. 1 **1977**, 789-795.
4. Kato, T.; Katagiri, N.; Wagai, A. Synthesis of methylpyridine derivatives-XXXIII Chem. Pharm. Bull. 25, 203 (1977): Phosphonylation and chlorination of methylpyridine and 3-nitro-methylpyridine derivatives. Tetrahedron **1978**, 34, 3445-3449.
5. Takahashi, K.; Mitsuhashi, K. Conversion of the carboxyl group to the corresponding trichloromethyl group in the quinoline series. J. Heterocycl. Chem. **1977**, 14, 881-884.
6. Conrad, M.; Limpach, L. Beiträge zur Kenntniss des γ-Oxychinaldins. Berichte **1888**, 21, 1965-1984.
7. Kato, T.; Katagiri, N.; Wagai, A. Trichloromethylquinolines: Synthesis and Reaction with Trimethyl Phosphite. Chem. Pharm. Bull. **1981**, 29, 1069-1075.
8. Baraldi, P. G.; Preti, D.; Tabrizi, M. A.; Fruttarolo, F.; Saponaro, G.; Baraldi, S.; Romagnoli, R.; Moorman, A. R.; Gessi, S.; Varani, K.; Borea, P. A. N6-[(Hetero)aryl/(cyclo)alkyl-carbamoyl-methoxy-phenyl]-(2-chloro)-5′-N-Ethylcarbox-amido-adenosines: The first example of adenosine-related structures with potent agonist activity at the human A2B adenosine receptor. Bioorg. Med. Chem. **2007**, 15, 2514-2527.
9. Galin, F. Z.; Sakhautdinov, I. M.; Tukhvatullin, O.R. Synthesis of pyrrolo[2,1-a]phthalazine-2,6-dione derivative from dioxophthalazine-containing sulfur ylide. Russ. Chem. Bull. **2007**, 56, 2305-2307.
